# Comprehensive Bioinformatic Investigation of TP53 Dysregulation in Diverse Cancer Landscapes

**DOI:** 10.3390/genes15050577

**Published:** 2024-04-30

**Authors:** Ruby Khan, Bakht Pari, Krzysztof Puszynski

**Affiliations:** 1 Department of Systems Biology and Engineering, Silesian University of Technology, 44-100 Gliwice, Poland; krzysztof.puszynski@polsl.pl; 2 Principal, Nursing School, Lady Reading Hospital Peshawar, Peshawar 25000, Pakistan; lalbahadar.lb@gmail.com

**Keywords:** p53 overexpression, cancer development, tumor progression, targeted therapy, TCGA database, disease-free survival, TP53 protein network

## Abstract

P53 overexpression plays a critical role in cancer pathogenesis by disrupting the intricate regulation of cellular proliferation. Despite its firmly established function as a tumor suppressor, elevated p53 levels can paradoxically contribute to tumorigenesis, influenced by factors such as exposure to carcinogens, genetic mutations, and viral infections. This phenomenon is observed across a spectrum of cancer types, including bladder (BLCA), ovarian (OV), cervical (CESC), cholangiocarcinoma (CHOL), colon adenocarcinoma (COAD), diffuse large B-cell lymphoma (DLBC), esophageal carcinoma (ESCA), head and neck squamous cell carcinoma (HNSC), kidney chromophobe (KICH), kidney renal clear cell carcinoma (KIRC), liver hepatocellular carcinoma (LIHC), lung adenocarcinoma (LUAD), lung squamous cell carcinoma (LUSC), and uterine corpus endometrial carcinoma (UCEC). This broad spectrum of cancers is often associated with increased aggressiveness and recurrence risk. Effective therapeutic strategies targeting tumors with p53 overexpression require a comprehensive approach, integrating targeted interventions aimed at the p53 gene with conventional modalities such as chemotherapy, radiation therapy, and targeted drugs. In this extensive study, we present a detailed analysis shedding light on the multifaceted role of TP53 across various cancers, with a specific emphasis on its impact on disease-free survival (DFS). Leveraging data from the TCGA database and the GTEx dataset, along with GEPIA, UALCAN, and STRING, we identify TP53 overexpression as a significant prognostic indicator, notably pronounced in prostate adenocarcinoma (PRAD). Supported by compelling statistical significance (*p* < 0.05), our analysis reveals the distinct influence of TP53 overexpression on DFS outcomes in PRAD. Additionally, graphical representations of overall survival (OS) underscore the notable disparity in OS duration between tumors exhibiting elevated TP53 expression (depicted by the red line) and those with lower TP53 levels (indicated by the blue line). The hazard ratio (HR) further emphasizes the profound impact of TP53 on overall survival. Moreover, our investigation delves into the intricate TP53 protein network, unveiling genes exhibiting robust positive correlations with TP53 expression across 13 out of 27 cancers. Remarkably, negative correlations emerge with pivotal tumor suppressor genes. This network analysis elucidates critical proteins, including SIRT1, CBP, p300, ATM, DAXX, HSP 90-alpha, Mdm2, RPA70, 14-3-3 protein sigma, p53, and ASPP2, pivotal in regulating cell cycle dynamics, DNA damage response, and transcriptional regulation. Our study underscores the paramount importance of deciphering TP53 dynamics in cancer, providing invaluable insights into tumor behavior, disease-free survival, and potential therapeutic avenues.

## 1. Background

The TP53 gene produces the p53 protein, which is essential for maintaining the integrity of cells. It controls a wide range of reactions to various stress signals, including metabolic stress, oncogene activation, and damage to DNA. By regulating several processes like cell cycle arrest, DNA repair, senescence, and apoptosis, p53 upholds genomic stability through its diverse regulatory roles [1,2,3]. All of these processes work together to provide a strong defense against malignant transformation.

The crucial function of p53 in maintaining cellular homeostasis and its significant consequences for the biology of cancer has been clarified by extensive research efforts. Significant research has revealed how the pathophysiology of many human malignancies is largely driven by the dysregulation of p53 activity, which is frequently triggered by TP53 gene mutations. Investigative pioneers Vogelstein, Minna, and associates [4,5,6] discovered the regular occurrence of extensive TP53 mutation databases [7,8,9,10], which provide additional confirmation of TP53 mutations in cancer.

These mutations, which are primarily found in the p53’s core DNA binding domain, cause the tumor suppressor protein to lose its function [1,11], which causes unchecked cell division, genomic instability, and the development of cancer. Interestingly, some missense mutations worsen tumorigenic processes by inhibiting wild-type p53 in a dominant-negative manner or by acquiring the characteristics of an oncogenic gain-of-function.

Moreover, abnormal p53 expression or activity has become a marker for aggressive tumor behavior, resistance to treatment, and poor clinical results in a wide range of cancer types, including gastrointestinal disorders, laryngeal squamous cell carcinoma, cervical and breast cancers, and others. This correlation highlights the complex relationship between p53 dysregulation and the advancement of cancer, hence requiring thorough research into the molecular mechanisms driving p53 malfunction.

Comprehending the complexities of p53 dysregulation has important therapeutic ramifications since it opens the door to the creation of focused therapies meant to restore p53 function and lessen the deleterious effects of cancer. Additionally, using integrated methods like The Cancer Genome Atlas Network (TCGA) provides a comprehensive understanding of the dynamics of TP53 mutations in a variety of cancer types, making it easier to identify new therapeutic targets and prognostic markers that will enhance patient outcomes and clinical management.

## 2. Introduction

Cancer represents a complex biological phenomenon characterized by dysregulated cell growth and survival [12]. Due to its biological roles, the p53 tumor suppressor is not only an important protector against malignant transformation but may also be used as a prognostic marker in cancer research [12]. As a transcription factor, p53 controls the growth of normal cells by coordinating the expression of genes that either promote cell cycle progression or cause cell cycle arrest in the G1 phase, especially in cases when the genome is damaged [13]. Furthermore, in response to DNA damage, active p53 is essential for inducing apoptosis in cells that have reached a growth halt [14]. This process is particularly effective when carcinogenic triggers are present. Recent studies have illuminated the intriguingly complex dynamics of p53 signaling, revealing not only its protective role in normal cells but also its paradoxical contributions to cancer pathogenesis when dysregulated. In cancer, TP53 is often mutated or expressed at abnormally high levels, altering p53’s normal regulatory functions and contributing to tumor progression and aggressiveness.

This dichotomy is apparent in a wide range of cancers, such as prostate adenocarcinoma, childhood malignant gliomas [15], endometrial carcinoma [16], and laryngeal squamous cell carcinoma [17], where overexpression of TP53 often predicts adverse outcomes. In these cancers, the status of TP53 is closely linked with disease progression and significantly affects patient outcomes. Moreover, theoretical models [18,19] have proposed that the dynamics of p53 in response to DNA damage, such as the oscillatory behavior driven by transcriptional and translational delays, are crucial for effective DNA repair. These models offer a potential window into manipulating p53 pathways therapeutically to balance its tumor-suppressing capabilities against its role in cancer promotion.

The fact that p53 is frequently mutated in around 50% of human malignancies indicates that p53 plays an important role in triggering cell cycle arrest or apoptosis progression [20,21,22]. When cells experience DNA damage, such as double-strand breaks (DSBs) brought on by ionizing radiation (IR) and other pharmaceutical agents, the p53 regulatory network is triggered. This arrests the cell cycle, allowing the cell to repair any damage, trigger transcription of a gene indirectly involved in DNA repair, and trigger apoptosis to eliminate irreversibly damaged cells [21,23,24]. Furthermore, Zhang et al. [25] suggested that the quantity of p53 pulses could predict a cell’s fate—whether it survives or dies. Additionally, Chen et al. [26] and Purvis et al. [27] suggested that p53 dynamics govern the DNA damage response, deciding a cell’s fate between life and death. These indicate that the development of cell control techniques requires a profound understanding of the dynamics of the p53 regulatory network in the DNA damage response.

The significance of p53 in cellular responses to genotoxic stimuli is highlighted by evidence suggesting that intact p53 can trigger apoptosis after exposure to ionizing radiation [28]. In contrast, p53 function loss has been reported linked to increased cellular resistance to a range of chemotherapeutic drugs, highlighting its function in regulating therapeutic responses during cancer treatment.

Gaining knowledge about the complex roles played by p53 might help us better understand cancer biology and may make it possible to use p53 status as a predictor [29] of treatment response and patient outcomes. This emphasizes how crucial it is to thoroughly examine TP53 mutations and the networks of interactions they have with other cancer types to understand the complex molecular mechanisms driving the development of cancer and to spot possible therapeutic weaknesses.

Although tremendous progress has been made in our understanding of the molecular causes of cancer, a substantial knowledge gap remains with regard to the thorough examination of TP53 mutations and the networks of interactions they entail in a variety of human malignancies. By using cutting-edge bioinformatic techniques to decipher the complex terrain of p53 changes across various cancer types, this study aims to close this gap. utilizing state-of-the-art instruments like GEPIA [30], UALCAN [31], and STRING [32], we aim to elucidate the molecular mechanisms underlying p53 dysregulation in cancer and identify novel therapeutic opportunities for personalized cancer management.

Prior research has established TP53 as one of the most frequently mutated genes in human cancers, with alterations in p53 function implicated in the initiation and progression of various malignancies. Furthermore, studies have consistently demonstrated an association between TP53 mutations and adverse clinical outcomes, including increased tumor aggressiveness and reduced patient survival [33,34,35]. However, despite this wealth of evidence, our understanding of the comprehensive landscape of TP53 mutations and their functional consequences across diverse cancer types remains incomplete.

While individual studies have provided valuable insights into the role of TP53 mutations in specific cancer contexts, a holistic analysis of TP53 alterations across a broad spectrum of human cancers is lacking. This knowledge gap represents a significant barrier to fully elucidating the molecular mechanisms driving cancer progression and limits the development of effective therapeutic strategies targeting p53 dysregulation. By conducting a comprehensive bioinformatic exploration of TP53 dynamics in diverse cancer landscapes, we aim to fill this critical void and uncover novel insights into the pathogenesis of cancer.

The lack of a comprehensive analysis of TP53 mutations and their associated interaction networks across various cancer types hinders our ability to decipher the molecular intricacies underlying cancer progression and identify potential therapeutic vulnerabilities. This knowledge deficit poses a significant challenge to the development of personalized cancer therapies tailored to the unique genetic and molecular characteristics of individual patients.

This study aims to conduct an exhaustive bioinformatic analysis of TP53 mutations and their related interaction networks across multiple human cancers, utilizing data and tools such as GEPIA [30], UALCAN [31], and STRING [32]. We hypothesize that a comprehensive exploration of TP53 dynamics in diverse cancer landscapes will unveil novel diagnostic and prognostic biomarkers, identify potential therapeutic targets, and deepen our understanding of the molecular mechanisms driving cancer development and progression. Statement of the problem: The lack of a comprehensive analysis of TP53 mutations and their associated interaction networks across various cancer types poses a formidable challenge to deciphering the molecular mechanisms driving cancer development and progression. This knowledge gap impedes the identification of potential therapeutic targets and undermines efforts to develop personalized cancer therapies customized to the unique characteristics of individual patients.

## 3. Materials and Methods

### 3.1. Data Sources

To perform our thorough research, we made use of a variety of bioinformatics tools and databases, including the following:

1. **GEPIA** [30]: With the use of this robust platform, we were able to conduct in-depth gene expression analyses across a variety of cancer types and normal tissues by having access to enormous datasets from the Genotype-Tissue Expression (GTEx) and the Cancer Genome Atlas (TCGA).
2. **UALCAN** [31]: By using this resource, we were able to examine TCGA gene expression data in more detail and gain insight into the ways that various cancer types express genes differently. This has allowed us to better understand cancer biology and identify possible targets for treatment.
3. **STRING Database** [32]: We used the STRING database to investigate the complex web of gene connections and protein–protein interactions. This made it easier for us to look into the biological importance and functional relationships of the genes linked to cancer pathways.

### 3.2. Analysis of TP53 Expression across Diverse Cancer Types

We examined TP53 expression in 27 different types of human tumors, representing a broad range of cancers. We conducted a thorough comparison of the TP53 expression levels in tumor tissues, comparable to normal tissues, and GTEx database data by utilizing the GEPIA online server. Adrenocortical Carcinoma (ACC), Bladder Urothelial Carcinoma (BLCA), Breast Invasive Carcinoma (BRCA), Esophageal Carcinoma (ESCA), Glioblastoma Multiforme (GBM), Head and Neck Squamous Cell Carcinoma (HNSC), Kidney Chromophobe (KICH), Kidney Renal Clear Cell Carcinoma (KIRC), Kidney Renal Papillary Cell Carcinoma (KIRP), Acute Myeloid Leukemia (LAML), Brain Lower Grade Glioma (LGG), Liver Hepatocellular Carcinoma (LIHC), Lung Adenocarcinoma (LUAD), Lung Squamous Cell Carcinoma (LUSC), Ovarian Serous Cystadenocarcinoma (OV), Pancreatic Adenocarcinoma (PAAD), Prostate Adenocarcinoma (PRAD), Uterine Corpus Endometrial Carcinoma (UCEC), Rectum Adenocarcinoma (READ), Skin Cutaneous Melanoma (SKCM), Stomach Adenocarcinoma (STAD), Testicular Germ Cell Tumors (TGCT), Thyroid Carcinoma (THCA), Thymoma (THYM), and Uterine Carcinosarcoma (UCS). The analysis did not include tumor forms with small sample sizes or without samples of normal tissue. Our statistical strategy included comparing comparable normal tissues, using log2(TPM + 1) transformed expression data, applying the ANOVA method to guarantee the robustness and dependability of our results for differential gene expression analysis, setting a strict *q*-value threshold of 0.01 and a |log2FC| threshold of 1.

### 3.3. Exploring Survival Dynamics

We examined TP53 expression-related survival outcomes in addition to assessing gene expression patterns. We examined overall survival (OS) and disease-free survival (DFS) results for different forms of cancer using the GEPIA web server. We determined hazard ratios (HR) using the Cox proportional hazards (PH) model and the Log-rank test to evaluate the effect of TP53 expression levels on patient survival. We thoroughly examined the differences in survival between high-expression and low-expression groups by stratifying patients based on a TP53 expression threshold of 50%. This allowed us to gain important insights into the prognostic importance of TP53 in various cancers.

### 3.4. Analyzing Gene Correlations: TP53’s Correlation Investigation

We explored the complex field of gene correlations, specifically aiming to clarify the connections between TP53 and various gene sets in various cancer types. Using paired examination of gene expression data obtained from the TCGA and GTEx databases, we clarified the extent and orientation of these correlations. Classified correlation coefficients provided insightful information on the TP53 network, allowing for the identification of genes that showed substantial correlations in each of the 27 cancer types and the fine-grained analysis of individual cancer subtypes. A greater comprehension of the molecular connections and regulatory mechanisms involving TP53 in the pathophysiology of cancer was made possible by this all-encompassing approach.

### 3.5. Constructing the TP53 Protein Network

By utilizing the STRING database to investigate functional protein association networks, our methodological framework resulted in the creation of the TP53 protein network. The network study included genes that showed substantial associations with TP53, as determined by the GEPIA web server. Important tumor suppressor proteins like ATR, ATM, BUB1B, BRCA1/2, CHK2, and CYLD were included in this extensive network, which provided insight into possible protein–protein interactions within the TP53 network. Our analysis untangled the complex network of molecular interactions, offering important new understandings of the functional consequences of TP53 dysregulation in cancer biology.

In conclusion, our methodology allowed for a thorough investigation of the critical function that TP53 plays in the pathophysiology of cancer, revealing the nuances of protein interactions, survival dynamics, and gene expression within complex cancer biology.

## 4. Results

### 4.1. Unraveling the Impact of P53 Overexpression in Tumors

P53, a pivotal tumor suppressor gene, orchestrates the delicate balance of the cell cycle, curbing uncontrolled cell division. However, elevated p53 expression can tip the scales, fostering the development and aggressive growth of tumors. This phenomenon is attributed to various factors, including exposure to carcinogens, genetic mutations, or viral infections. The scope of p53 overexpression extends across a spectrum of cancers, encompassing bladder, ovarian, cervical, lung, and breast cancers. Notably, heightened p53 expression is associated with more aggressive cancers, carrying a higher risk of recurrence. Treatment strategies for tumors exhibiting p53 overexpression often involve a multifaceted approach, combining targeted therapies directed at the p53 gene with chemotherapy, radiation therapy, and targeted drug therapies.

**Figure 1:**
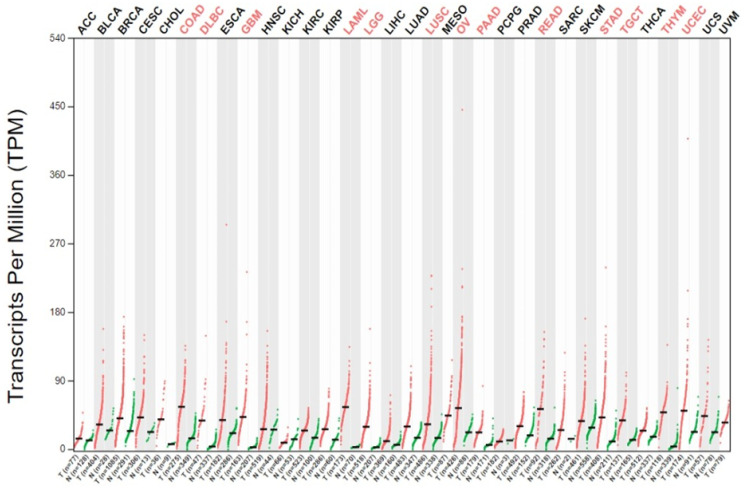
Exploring the TP53 gene expression profile across TCGA tumors.

Figure 1 offers a glimpse into the dynamic expression patterns of the TP53 gene across 13 diverse TCGA tumors out of the 27 analyzed, comparing tumor samples with their normal counterparts and GTEx data. The vivid representation showcases significant TP53 overexpression in 13 out of the 27 cancers, each TCGA tumor highlighted in red, while matched normal and GTEx data are delineated in green. The *Y*-axis reflects transcript per million (log2(TPM)), and the *X*-axis portrays the count of tumor and normal samples.

The specific cancers analyzed include ACC, BLCA, BRCA, COAD, DLBC, ESCA, GBM, HNSC, KICH, KIRC, KIRP, LAML, LGG, LIHC, LUAD, LUSC, OV, PAAD, PRAD, READ, SKCM, STAD, TGCT, THCA, THYM, UCEC, and UCS.

**Figure 2:**
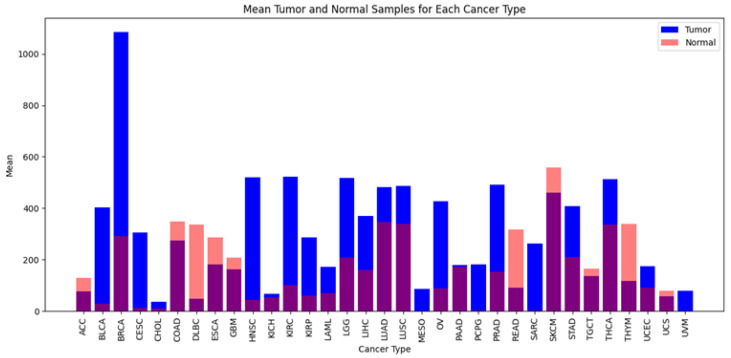
Comparison of mean tumor and normal samples across different cancer types.

In Figure 2, The bar plots display the expression levels of TP53 in tumor (T) and normal (N) cells across various cancer types, focusing on those where TP53 is overexpressed. In the bar plots, the number of tumor samples (T) is compared to the number of normal samples (N) for each cancer type. The cancer types where TP53 is overexpressed include (1) Bladder Urothelial Carcinoma (BLCA), (2) Breast Invasive Carcinoma (BRCA), (3) Cervical Squamous Cell Carcinoma and Endocervical Adenocarcinoma (CESC), (4) Colon Adenocarcinoma (COAD), (5) Diffuse Large B-cell Lymphoma (DLBC), (6) Esophageal Carcinoma (ESCA), (7) Glioblastoma Multiforme (GBM), (8) Head and Neck Squamous Cell Carcinoma (HNSC), (9) Kidney Renal Clear Cell Carcinoma (KIRC), (10) Kidney Renal Papillary Cell Carcinoma (KIRP), (11) Liver Hepatocellular Carcinoma (LIHC), and (12) Lung Adenocarcinoma (LUAD). These cancer types demonstrate a higher number of tumor samples compared to normal samples, suggesting potential overexpression of TP53, which may contribute to the pathogenesis of these cancers. Additionally, Rectum Adenocarcinoma (READ) demonstrates a higher number of tumor samples relative to normal samples, suggesting potential overexpression of TP53 in rectum adenocarcinoma.

### 4.2. Mapping P53’s Journey through Pathological Stages

P53’s influence transcends through five distinct pathological stages, each categorized based on p53 expression levels within tumors. Stage I exhibits minimal p53 expression, while stage V presents the highest levels. Tumors with heightened p53 expression often manifest malignant characteristics, carrying a poorer prognosis. Therapeutic approaches for such cases involve targeted therapies directed at the p53 gene, complemented by chemotherapy, radiation therapy, and targeted drug therapies.

### 4.3. Decoding P53 Subtypes

P53 manifests in four distinct subtypes, shaped by genetic mutations or alterations: wild-type, mutant, overexpressed, and deleted. The wild-type represents the normal gene form, while mutant, overexpressed, and deleted forms signify genetic deviations. Each subtype aligns with different cancer types, warranting tailored treatment strategies. For instance, mutant p53 is associated with specific lung cancers, suggesting targeted therapies as potential interventions. Overexpressed p53, linked to certain breast cancers, may find remediation through a combination of chemotherapy, radiation, and targeted drug therapies.

### 4.4. Exploring the Nexus of P53 Overexpression with Histological and Molecular Subtypes

Our investigation delves into the intricate interplay of p53 overexpression across diverse histological and molecular subtypes of cancer, unraveling significant findings as presented in Table 1. Leveraging data from the TCGA database, our analysis reveals a notable upregulation of TP53 expression in 12 out of 27 tumor types when compared with corresponding TCGA normal tissues and GTEx data (Figure 1). Further scrutiny involves a comprehensive assessment of TP53 expression within normal tissues, employing RNA-sequencing data from the GTEx dataset. The results illuminate heightened TP53 expression in 12 distinct cancer types compared to normal tissues, as vividly depicted in the individual boxplot representations below.

Our exploration of TP53 expression extends across various dimensions, including tumor molecular and histological subtypes, tumor grades, and patient conditions, all scrutinized through the lens of the UALCAN tool. Noteworthy among our findings is the overexpression observed in 13 out of 27 cancers, including COAD, DLBC, GBM, LAML, LGG, LUSC, OV, PAAD, READ, STAD, TGCT, THYM, and UCEC.

In urologic cancers, heightened TP53 expression takes center stage in BLCA histological subtypes, particularly in papillary and non-papillary tumors, surpassing normal samples (Table 1). Molecular subtypes within BLCA exhibit elevated expression, notably in the luminal papillary subtype and basal squamous subtype (Table 1). KIRC showcases increased TP53 expression across all tumor grades, with heightened significance in grade 2 tumors. PRAD, on the other hand, manifests its most significant overexpression in Gleason score 8, with notable shifts observed in ETS transcription factor ERG fusion.

Within BRCA tumors, the dominance of infiltrating ductal carcinoma (IDC) stands out with statistically significant overexpression compared to normal tissue, supported by compelling evidence from molecular subtypes, patient conditions, and tumor stage, all underscored by noteworthy *p*-values below 0.05.

COAD analysis reveals striking disparities in gene expression between normal tissue and adenocarcinoma, hinting at profound clinical implications worthy of further exploration, considering the molecular subtypes, other patient conditions, and tumor stage.

ESCA analysis uncovers intricate correlations between TP53 expression and various subtypes, grades, molecular subtypes, and other conditions associated with normal tissue, shedding light on the complex interplay within the esophageal cancer landscape.

HNSC investigation furnishes valuable insights into TP53 expression patterns across diverse histological subtypes, grades, other patient conditions, and tumor stages, offering a nuanced understanding of its role in head and neck cancer progression.

KIRC showcases a consistent elevation in TP53 expression across all tumor grades, with particular emphasis on specific molecular subtypes, other patient conditions, and tumor stages, unveiling potential avenues for targeted therapeutic interventions.

KIRP analysis delineates notable differences in TP53 expression across different histological and molecular subtypes, tumor grades, other patient conditions, and tumor stages, providing crucial insights into the heterogeneous nature of kidney renal papillary cell carcinoma.

LIHC examination uncovers compelling associations between TP53 expression and distinct tumor grades, other patient conditions, and tumor stage, highlighting its clinical relevance in liver hepatocellular carcinoma progression and prognosis.

READ analysis accentuates substantial differences in TP53 expression across various histological subtypes, other patient conditions, and tumor stages, offering valuable insights for tailored treatment strategies.

PAAD analysis elucidates significant associations between TP53 expression and various drinking habits, other patient conditions, and tumor stage, suggesting potential implications for personalized patient management in pancreatic adenocarcinoma.

LGG scrutiny reveals significant differences in TP53 expression across diverse histological subtypes, tumor grades, other patient conditions, and tumor stages, underscoring its multifaceted role in glioma biology.

LUAD analysis furnishes valuable insights into TP53 expression patterns amidst different histological subtypes, other patient conditions, and tumor stages, offering critical knowledge for precision medicine approaches in lung adenocarcinoma treatment.

LUSC examination unravels significant associations between TP53 expression and various histological subtypes, other patient conditions, and tumor stages, enriching our understanding of its involvement in lung squamous cell carcinoma progression.

OV analysis showcases marked differences in TP53 expression across different tumor grades, other patient conditions, and tumor stages, providing key insights into its role in ovarian cancer development and prognosis.

PRAD analysis underscores significant associations between TP53 expression, Gleason score, molecular subtypes, other patient conditions, and tumor stage, offering valuable prognostic markers for prostate adenocarcinoma.

STAD analysis offers intricate insights into diverse aspects of the disease, unraveling significant associations between TP53 expression and various subtypes, grades, other patient conditions, and tumor stages, illuminating the complex landscape of stomach  adenocarcinoma.

TGCT analysis unveils substantial differences in TP53 expression between seminoma and non-seminoma subtypes, other patient conditions, and tumor stage, shedding light on its role in testicular germ cell tumor biology.

THYM examination elucidates profound distinctions between different types and subtypes, providing invaluable insights for diagnostic and therapeutic strategies in thymoma, considering other patient conditions and tumor stages.

UCEC analysis unravels exceptional significance in various comparisons, indicating substantial differences with profound clinical implications for uterine corpus endometrial carcinoma, including other patient conditions and tumor stages.

UCS analysis provides illuminating insights into TP53 expression patterns across diverse histological subtypes, other patient conditions, and tumor stages, enriching our understanding of its involvement in uterine carcinosarcoma progression.

In conclusion, our findings underscore the complex and heterogeneous nature of TP53 expression patterns across diverse cancer types, emphasizing the necessity for nuanced analyses to comprehensively grasp the clinical and biological relevance of these findings. The statistical comparisons provided offer a detailed perspective on the associations within specific cancer types, enriching the broader landscape of cancer research and paving the way for targeted therapeutic interventions and precision medicine approaches in oncology.

As presented in Table 2, this table showcases distinctive genes associated with a variety of cancer types.

#### Unraveling TP53 Gene and Biological Pathways

In our investigation, we delved into the biological pathways connected to the TP53 gene, commonly known as the p53 gene—a pivotal regulator governing diverse cellular processes. These pathways were unearthed through the KEGG (Kyoto Encyclopedia of Genes and Genomes) database, offering profound insights into how TP53 influences various facets of cell biology and its implications for cancer.

- **p53 Signaling Pathway (hsa04115):** Central to TP53’s function, this pathway involves crucial proteins such as p53 apoptosis effector related to PMP22 (PERP) and Tumor protein p53 inducible protein 11 (TP53I11), pivotal in mediating TP53’s impact on cell survival, DNA repair, and apoptosis.
- **Endocrine Resistance (hsa01522):** Unveiling a connection between TP53 and cytochrome P450 family 2 subfamily D member 6, our study suggests a potential role for TP53 in endocrine resistance, particularly within the context of cancer therapy.
- **Platinum Drug Resistance (hsa01524):** Our findings shed light on TP53’s involvement in platinum drug resistance, a formidable challenge in cancer treatment. Proteins like ERCC1, MLH1, MSH2, and GSTP1 play pivotal roles in this resistance mechanism.
- **MAPK Signaling Pathway (hsa04010):** TP53 appears to engage with proteins associated with the MAPK signaling pathway, regulating cell growth and differentiation. This interaction hints at a broader role for TP53 in cellular responses to external signals.
- **Ras Signaling Pathway (hsa04014):** Within this pathway, TP53 interacts with proteins such as RAS, RAF, and MEK, suggesting its potential involvement in the regulation of cell proliferation and growth, particularly in the context of cancer.
- **Cell Cycle (hsa04110):** Our exploration supports the well-established role of TP53 in governing the cell cycle. Proteins like BUB1B, BUBR1, MAD3L, and others contribute to TP53-mediated control across various phases of the cell cycle.
- **PI3K-Akt Signaling Pathway (hsa04151):** TP53’s participation in the PI3K-Akt signaling pathway implies its role in cell survival and proliferation, potentially influencing cancer progression.
- **Apoptosis (hsa04210):** Recognized for its critical role in apoptosis, TP53’s association with proteins in the extrinsic apoptotic pathway enhances our understanding of how TP53 regulates programmed cell death.
- **Pathways in Cancer (hsa05200):** TP53’s involvement in diverse signaling cascades, including EGF-EGFR-RAS-ERK, underscores its significance in the development and progression of cancer. These pathways offer a comprehensive view of TP53’s contributions to cancer-related processes.
- **Ferroptosis (hsa04216):** Our investigation suggests that TP53 may influence ferroptosis, a regulated cell death process, adding to our comprehension of TP53’s role in cell fate decisions.
- **Cellular Senescence (hsa04218):** TP53’s presence in the cellular senescence pathway highlights its role in driving cells into a state of irreversible growth arrest—an essential mechanism to prevent uncontrolled cell division.
- **Ubiquitin-Mediated Proteolysis (hsa04120):** TP53’s association with proteins like BRCA1, CDC20, UBE2C, and UBE2S underscores its involvement in protein degradation, providing insights into how TP53 regulates the turnover of key cellular proteins.

These findings collectively unveil the multifaceted regulatory role of TP53 across diverse cellular processes and its pervasive engagement in the pathways associated with cancer. Comprehending these intricate connections provides valuable insights into the intricate biology of TP53, unraveling its implications in the realms of cancer development, drug resistance, and other nuanced cellular responses.

Table 3 shows the expression based on TP53 in mutation comparison for various cancer types.

In Table 4, we present a comprehensive overview of the various biological pathways associated with TP53, which is a pivotal gene in cancer biology. It plays a significant role in regulating cell cycle arrest, DNA repair, apoptosis, and more. The table provides insights into how TP53 is interconnected with other critical proteins and pathways in the cell.

Additionally, for a detailed examination of the proteins involved in the p53 signaling pathway, you can refer to Table 4. This subtable further delves into the intricate network of serine/threonine kinases, receptor kinases, and tyrosine kinases, shedding light on the molecular intricacies of this crucial cellular response system.

### 4.5. Survival Analysis Figures

**Figure 3:**
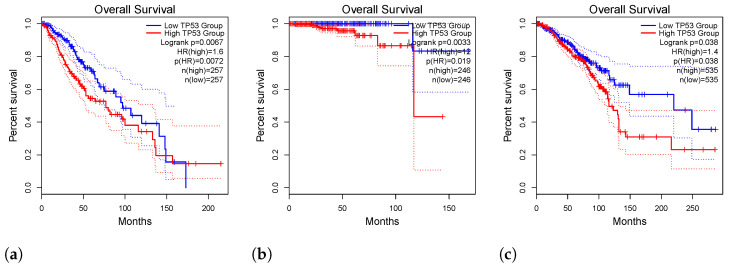
Survival analyses for LGG, PRAD, and BRCA. (**a**) Survival analysis for LGG (Low-Grade Glioma). (**b**) Survival analysis for PRAD (Prostate Adenocarcinoma). (**c**) Survival analysis for BRCA (Breast Invasive Carcinoma).

Figure 3a: This figure depicts the results of a survival analysis conducted for Low-Grade Glioma (LGG), elucidating the prognosis of patients afflicted with this particular type of brain tumor. The insights gleaned from this analysis offer valuable information regarding the survival outcomes and trends associated with LGG.

Figure 3b: Presented here is a survival analysis focused on Prostate Adenocarcinoma (PRAD), providing essential data on the survival outcomes for individuals diagnosed with this variant of prostate cancer. The findings presented in this figure contribute significantly to our understanding of the prognosis and survival rates in PRAD patients.

Figure 3c: In this figure, we observe the survival analysis conducted for Breast Invasive Carcinoma (BRCA), offering insights into the survival rates and trends within the realm of breast cancer. The data presented herein shed light on crucial factors influencing the prognosis and survival outcomes of patients with BRCA.

**Figure 4:**
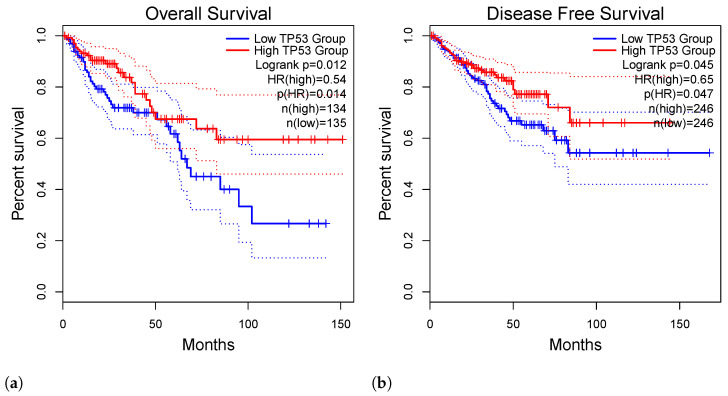
Survival analysis for COAD and disease-related analysis for PRAD. (**a**) Survival analysis for COAD (Colon Adenocarcinoma). (**b**) Disease-related analysis for PRAD (Prostate Adenocarcinoma).

Figure 4a: This figure showcases a survival analysis dedicated to Colon Adenocarcinoma (COAD), providing critical insights into the survival prospects of individuals afflicted with colon cancer. The findings presented in this figure contribute substantially to our understanding of the factors influencing survival outcomes in COAD patients.

Figure 4b: Within this figure, we delve into a disease-related analysis specifically tailored for Prostate Adenocarcinoma (PRAD), offering a comprehensive examination of factors impacting the progression and severity of the disease. The insights derived from this analysis are essential for elucidating the underlying mechanisms driving PRAD and guiding therapeutic interventions.

**Figure 5:**
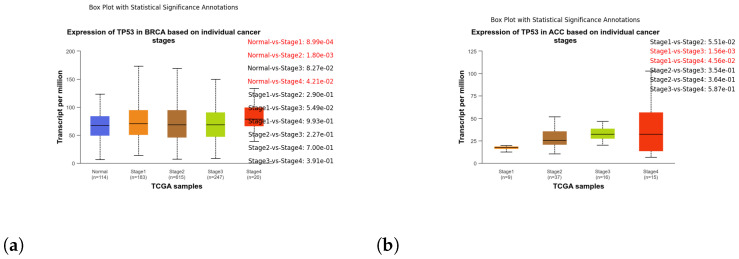
Comparative analysis of TP53 expression in Brain Tissue (BRCA) and Adenoid Cystic Carcinoma (ACC) stages: box plot visualization. (**a**) Box plot illustrating the expression of TP53 in different stages of brain tissue. (**b**) Box plot illustrating the expression of TP53 in stages of ACC.

**Figure 6:**
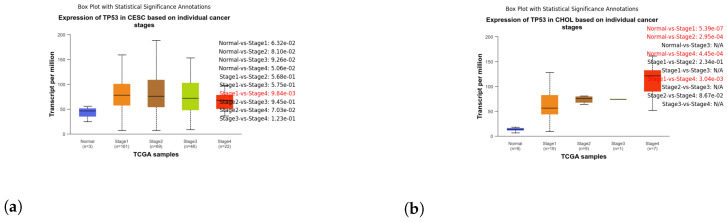
Exploring TP53 expression patterns in Cervical Squamous Cell Carcinoma (CESC) and Cholangiocarcinoma (CHOL) progression: box plot analysis. (**a**) Box plot illustrating the expression of TP53 in different stages (CESC). (**b**) Box plot illustrating the expression of TP53 in stages of CHOL.

**Figure 7:**
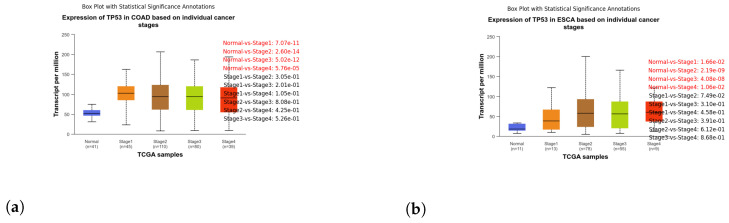
Analyzing TP53 expression dynamics in Colorectal Adenocarcinoma (COAD) and Esophageal Carcinoma (ESCA) progression: a box plot examination. (**a**) Box plot illustrating the expression of TP53 in stages of COAD. (**b**) Box plot illustrating the expression of TP53 in stages of ESCA.

**Figure 8:**
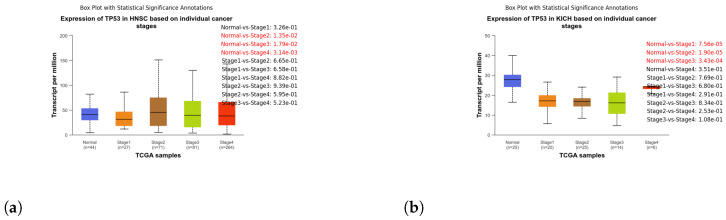
Box plot analysis depicting TP53 expression dynamics in stages of Head and Neck Squamous Cell Carcinoma (HNSC) and Kidney Chromophobe (KICH) tumorigenesis. (**a**) Box plot illustrating the expression of TP53 in stages of HNSC. (**b**) Box plot illustrating the expression of TP53 in stages of KICH.

**Figure 9:**
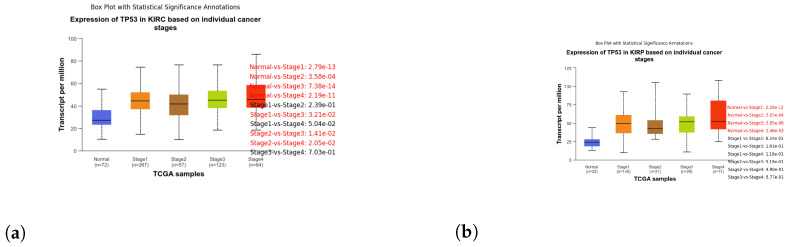
Box plots depicting the expression of TP53 in various stages of Kidney Renal Clear Cell Carcinoma (KIRC) and Kidney Renal Papillary Cell Carcinoma (KIRP), shedding light on the differential expression patterns across tumor stages. (**a**) Box plot illustrating the expression of TP53 in stages of KIRC. (**b**) Box plot illustrating the expression of TP53 in stages of KIRP.

**Figure 10:**
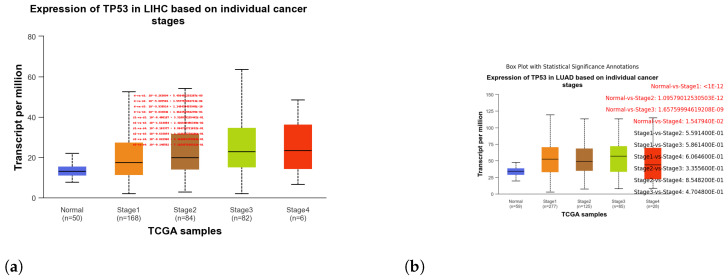
Box plots showcasing the expression levels of TP53 across different stages of Liver Hepatocellular Carcinoma (LIHC) and Lung Adenocarcinoma (LUAD), providing insights into the variations in TP53 expression throughout tumor progression. (**a**) Box plot illustrating the expression of TP53 in stages of LIHC. (**b**) Box plot illustrating the expression of TP53 in stages of LUAD.

**Figure 11:**
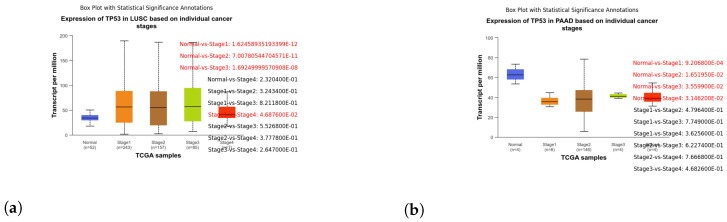
Analysis of TP53 expression across stages of Lung Squamous Cell Carcinoma (LUSC) and Pancreatic Adenocarcinoma (PAAD) using box plots. (**a**) Box plot illustrating the expression of TP53 in stages of LUSC. (**b**) Box plot illustrating the expression of TP53 in stages of PAAD.

**Figure 12:**
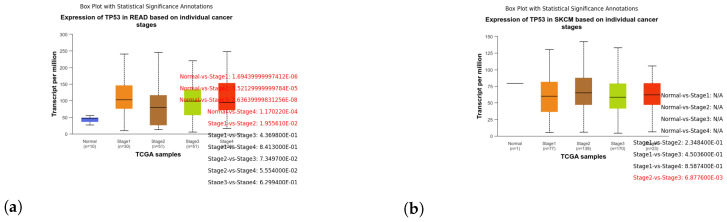
Exploring TP53 expression patterns in Rectum Adenocarcinoma (READ) and Skin Cutaneous Melanoma (SKCM) through box plot analysis. (**a**) Box plot illustrating the expression of TP53 in stages of READ. (**b**) Box plot illustrating the expression of TP53 in stages of SKCM.

**Figure 13:**
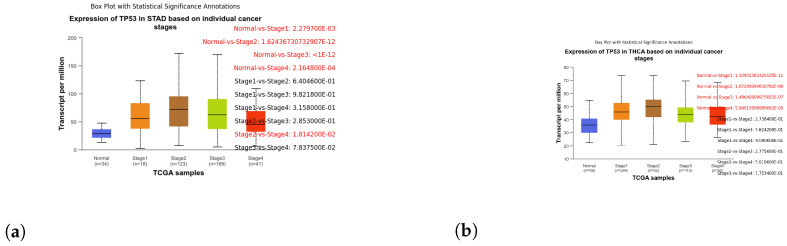
Characterizing TP53 expression patterns in Stomach Adenocarcinoma (STAD) and Thyroid Carcinoma (THCA) using box plot analysis. (**a**) Box plot illustrating the expression of TP53 in stages of STAD. (**b**) Box plot illustrating the expression of TP53 in stages of THCA.

**Figure 14:**
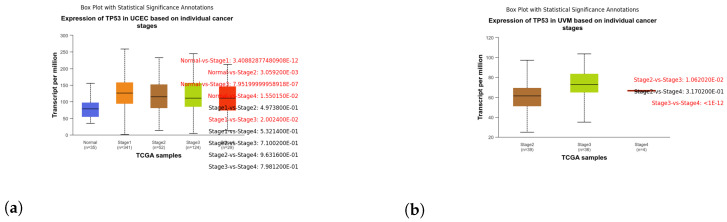
Analyzing TP53 expression variations in Uterine Corpus Endometrial Carcinoma (UCEC) and Uveal Melanoma (UVM) through box plot analysis. (**a**) Box plot illustrating the expression of TP53 in stages of UCEC. (**b**) Box plot illustrating the expression of TP53 in stages of UVM.

These box plots illustrate the expression of TP53 in different cancer types. Brain tissue (BRCA) Figure 5a, Adenoid cystic carcinoma (ACC) Figure 5b, Cervical squamous cell carcinoma and endocervical adenocarcinoma (CESC) Figure 6a, Cholangiocarcinoma (CHOL) Figure 6b, Colon adenocarcinoma (COAD) Figure 7a, Esophageal carcinoma (ESCA) Figure 7b, Head and neck squamous cell carcinoma (HNSC) Figure 8a, Kidney chromophobe (KICH) Figure 8b, Kidney renal clear cell carcinoma (KIRC) Figure 9a, Kidney renal papillary cell carcinoma (KIRP) Figure 9b, Liver hepatocellular carcinoma (LIHC) Figure 10a, Lung adenocarcinoma (LUAD) Figure 10b, Lung squamous cell carcinoma (LUSC) Figure 11a, Pancreatic adenocarcinoma (PAAD) Figure 11b, Rectum adenocarcinoma (READ) Figure 12a, Skin cutaneous melanoma (SKCM) Figure 12b, Stomach adenocarcinoma (STAD) Figure 13a, Thyroid carcinoma (THCA) Figure 13b, Uterine corpus endometrial carcinoma (UCEC) Figure 14a, and Uveal melanoma (UVM) Figure 14b. These box plots depict the distribution of TP53 expression levels across various cancer types and stages. Statistical significance annotations have been added to the plots, indicating the significance of pairwise comparisons between these stages. Each annotation includes a comparison label and the corresponding *p*-value. Annotations colored in red denote statistically significant differences (p≤0.05), while black annotations indicate non-significant differences (p>0.05). These annotations serve to elucidate the biological significance of TP53 expression variations within each cancer type.

In the realm of advanced oncology, our investigation delved into the nuanced role of TP53 overexpression in shaping cancer prognosis, employing meticulous analysis of The Cancer Genome Atlas (TCGA) data across various tumor types. The study aimed to unravel the intricate relationships between TP53 gene expression levels and clinical outcomes.

TP53 Overexpression and Overall Survival (OS): Elevated expression of TP53 has been identified as a significant determinant affecting overall survival in specific malignancies. Patients exhibiting heightened TP53 levels experienced notably shorter overall survival (OS) and a substantially unfavorable prognosis in cancers such as BRCA, LGG, LUAD, PRAD, COAD, and SKCM (refer to Figure 3a–c and Figure 4a). The statistical significance of these associations (*p*-value < 0.05) underscores the central role of TP53 in influencing the clinical outcomes of these cancers. Detailed analyses of other malignancies with *p*-values above 0.05 are provided in the Supplementary File for further exploration.

TP53 Overexpression and Disease-Free Survival (DFS): Furthermore, our investigation extended to disease-free survival (DFS), uncovering TP53 overexpression as a predictor of poorer prognoses, notably observed in PRAD (refer to Figure 4b). This unique yet compelling finding, bolstered by significant changes (*p*-value < 0.05), underscores the distinct impact of TP53 overexpression on DFS outcomes in prostate cancer. Detailed analyses of DFS for other cancers with *p*-values above 0.05 are available in the Supplementary File for comprehensive exploration.

Broad Impact Across Tumor Types: TP53 overexpression has demonstrated a profound impact on overall survival across various tumor types. Patients with heightened TP53 expression experienced significantly shorter overall survival and notably unfavorable prognoses in cancers including BLCA, BRCA, CESC, CHOL, COAD, DLBC, ESCA, HNSC, KICH, KIRC, LIHC, LUAD, LUSC, UCEC and a few others, as mentioned above (refer to Figure 5, Figure 6, Figure 7, Figure 8, Figure 9, Figure 10, Figure 11, Figure 12, Figure 13 and Figure 14). The significance of these findings (*p*-value < 0.05) underscores the adverse clinical implications associated with TP53 overexpression across a diverse spectrum of cancer types. Specifically, we have focused on individual stages within each cancer where the *p*-value is either equal to or below 0.05. Additionally, for each figure a corresponding table has been included, featuring only those comparisons between normal versus stages or stages versus stages that meet our specified criteria.

In essence, our study sheds light on the multifaceted impact of TP53 overexpression on survival outcomes, emphasizing its role as a critical determinant in the clinical trajectory of specific cancers. These findings contribute valuable insights to the broader understanding of TP53-associated prognostic implications in oncology.

The graphical representations in overall survival analysis figures vividly illustrate the stark contrast in OS time between tumors with higher TP53 expression (depicted by the red line) and those with lower TP53 expression (indicated by the blue line). The hazard ratio (HR) further underscores the impact of TP53 on overall survival.

In summary, our comprehensive analysis provides compelling evidence of the pivotal roles played by TP53 and TP53 overexpression in shaping cancer prognosis across various tumor types. These findings carry significant implications for clinical management and underscore the need for further research into the underlying molecular mechanisms driving these associations.

#### p53 Protein Network

Genes with strong or very strong positive correlations with TP53 expression in 13 among 27 cancers and also some negative correlations with important tumor suppressor genes were identified in the TP53 protein network.

The protein deacetylase sirtuin-1 (SIRT1) is a crucial enzyme involved in regulating various cellular functions. It serves as an NAD-dependent protein deacetylase, linking transcriptional regulation to intracellular energetics. SIRT1 plays a pivotal role in coordinating cellular processes such as the cell cycle, DNA damage response, metabolism, apoptosis, and autophagy. One of its primary functions is the deacetylation of histones, which can modify chromatin function, leading to transcriptional repression. Additionally, SIRT1 can deacetylate a broad range of transcription factors and coregulators, thus regulating the expression of target genes.

CREB-binding protein (CBP) is an acetyltransferase that plays a key role in histone acetylation. By acetylating histones, CBP adds specific tags for transcriptional activation. Furthermore, CBP can acetylate non-histone proteins like DDX21, FBL, IRF2, MAFG, NCOA3, POLR1E/PAF53, and FOXO1. It specifically binds to phosphorylated CREB, enhancing its transcriptional activity towards cAMP-responsive genes. CBP also acts as a coactivator of various transcriptional activators, including the NPAS2-ARNTL/BMAL1 and CLOCK-ARNTL/BMAL1 heterodimers involved in circadian rhythm regulation.

Histone acetyltransferase p300 is another important enzyme involved in histone acetylation and transcriptional regulation. p300 acetylates all four core histones in nucleosomes, resulting in an epigenetic mark that promotes transcriptional activation. It mediates cAMP-gene regulation by binding to phosphorylated CREB protein and acetylates histone H3 at specific lysine residues, including ‘Lys-122’ and ‘Lys-27’, contributing to transcriptional stimulation.

Serine-protein kinase ATM is a crucial sensor of DNA damage and cellular stress. It activates checkpoint signaling pathways in response to double-strand breaks, apoptosis, and genotoxic stresses. ATM phosphorylates ‘Ser-139’ of histone variant H2AX at double-strand breaks, thereby regulating the DNA damage response. It also plays a role in processes such as pre-B cell allelic exclusion, which enforces clonality and monospecificity of immunoglobulin heavy chain alleles.

Death domain-associated protein 6 (DAXX) functions as a transcription corepressor, repressing the transcriptional potential of sumoylated transcription factors. DAXX can inhibit basal and activated transcription by modulating subnuclear compartments like the nucleolus or PML/POD/ND10 nuclear bodies. It may influence TNFRSF6-dependent apoptosis and inhibit the transcriptional activation of specific genes, such as PAX3 and ETS1.

Heat shock protein HSP 90-alpha is a molecular chaperone responsible for promoting the maturation, structural maintenance, and regulation of various target proteins. It engages in a functional cycle linked to its ATPase activity, facilitating the activation of client proteins. HSP 90-alpha interacts dynamically with co-chaperones to modulate substrate recognition and chaperone function.

E3 ubiquitin-protein ligase Mdm2 is a critical regulator of p53 protein levels. It mediates the ubiquitination of p53, leading to its degradation by the proteasome. Mdm2 also inhibits the transcriptional activation of p53 by binding to its activation domain and promotes the nuclear export of p53. Additionally, it plays a role in various cellular processes, including cell cycle regulation and apoptosis.

Replication protein A 70 kDa DNA-binding subunit (RPA70) is a component of the RPA complex, which stabilizes single-stranded DNA intermediates during DNA replication and DNA stress. RPA70 prevents the re-annealing of single-stranded DNA and recruits the various proteins involved in DNA metabolism. It plays a crucial role in DNA replication and the cellular response to DNA damage.

14-3-3 protein sigma is an adapter protein involved in the regulation of multiple signaling pathways. It binds to phosphorylated serine or threonine motifs in various partner proteins, modulating their activity. 14-3-3 protein sigma can regulate protein synthesis, epithelial cell growth, and the degradation of MDM2, leading to the activation of p53.

Cellular tumor antigen p53 (p53) is a well-known tumor suppressor that regulates cell cycle progression and apoptosis. It acts as a trans-activator to negatively regulate cell division by controlling a set of genes essential for this process. p53 can induce growth arrest or apoptosis in response to different cellular circumstances, depending on the context. It plays a central role in preventing the development of various tumor types.

Apoptosis-stimulating of p53 protein 2 (ASPP2) is a regulator that interacts with TP53 (p53) and enhances its DNA binding and transactivation function. ASPP2 is involved in the regulation of apoptosis and cell growth and can inhibit cell cycle progression at G2/M. It also plays a role in modulating the activity of proteins like APPBP1 and DDX42.

The mentioned proteins in the TP53 network are associated with strong or very strong positive correlations with TP53 expression in various cancers. They also participate in different cellular pathways, including those related to cell cycle regulation, DNA damage response, and transcriptional control. Furthermore, proteins with TF binding sites on both the promoter and enhancer regions of UBE2C are involved in the TP53 network, contributing to the complexity of its regulatory interactions. TSPYL2, a tumor suppressor protein, interacts with UBE2C and is implicated in chromatin remodeling and RNA-RNA and RNA-protein interactions in the context of the UBE2C protein network. Additionally, the presence of a D-box motif in TSPYL2 suggests its involvement in the ubiquitin–proteasome pathway. These findings highlight the intricate network of protein–protein and protein–RNA interactions in the regulation of cellular processes associated with TP53.

**Figure 15:**
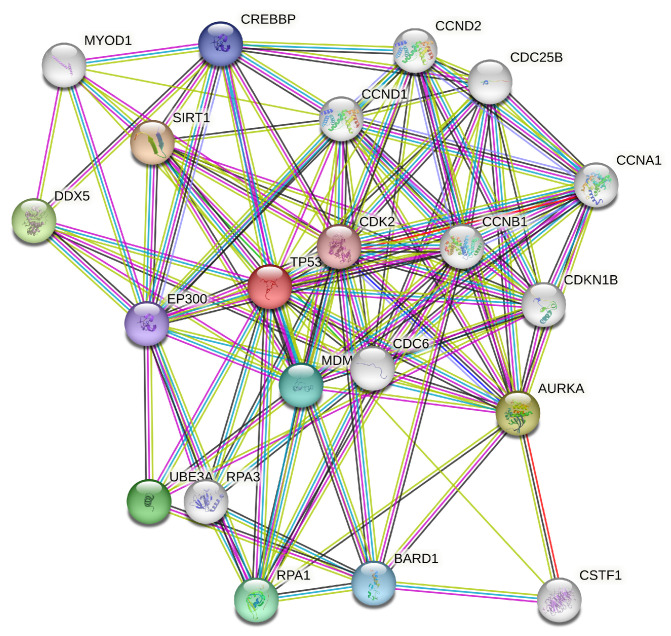
TP53 protein network from STRING database.

In Figure 15, we are presented with an illustration depicting the intricate TP53 protein network sourced from the comprehensive STRING database. This visual representation offers a glimpse into the complex interplay of TP53 proteins and their interactions as documented within the STRING database.

**Figure 16:**
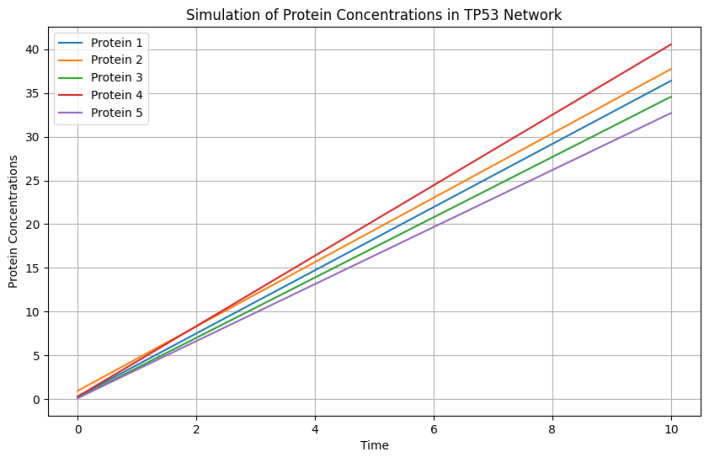
Simulation of protein concentrations.

In Figure 16, protein concentrations in a TP53 network are simulated over time (0 to 10 units) for n=5 proteins and m=27 cancer types. The ODE system, defined by the model function, integrates numerical solutions for protein concentration profiles.

## 5. Additional Analyses

### 5.1. Steady-State Analysis

**Figure 17:**
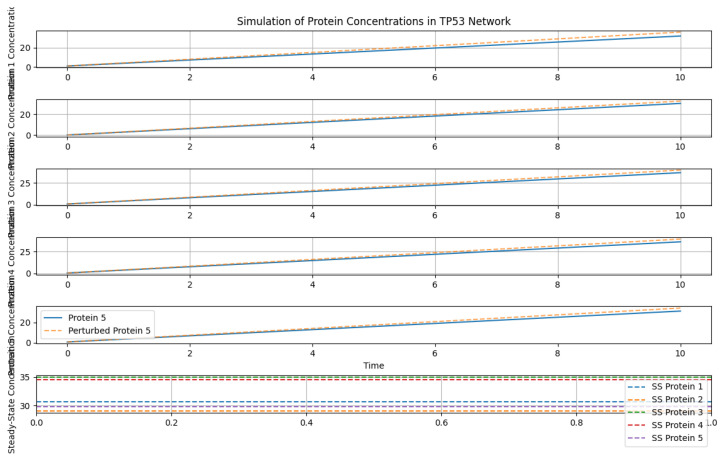
Steady-state analysis.

In Figure 17, the steady-state concentrations (SS) for each protein were calculated as the mean over the last 10 time units. Dashed lines were included in the plot to represent steady-state concentrations, aiding in the identification of proteins reaching stability. This analysis provides valuable information about the equilibrium levels of proteins within the TP53 network, shedding light on their potential roles in cellular processes.

### 5.2. Sensitivity Analysis

**Figure 18:**
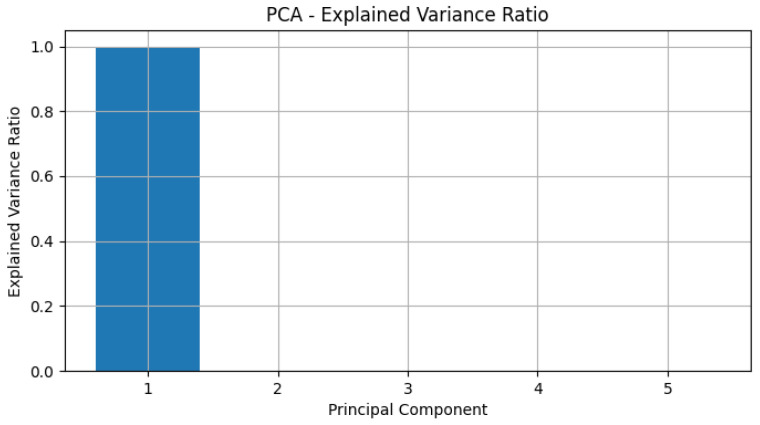
Sensitivity analysis.

Figure 18 illustrates the sensitivity analysis conducted by perturbing rate constants with 10% random noise. Solid lines depict original concentrations, while dashed lines represent perturbed concentrations, exploring the impact of rate constant changes on protein concentrations. This analysis helps assess the robustness of the TP53 network to variations in kinetic parameters, providing insights into its stability and responsiveness to external stimuli.

### 5.3. Parameter Sweep Analysis

**Figure 19:**
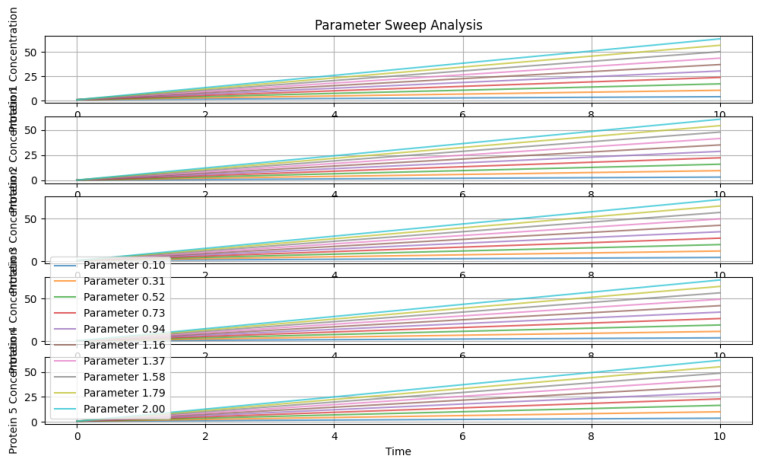
Parameter sweep analysis.

In Figure 19, a parameter sweep analysis was performed by varying values from 0.1 to 2.0, scaling rate constants. Subplots display protein concentration profiles for different parameter values, revealing the system’s sensitivity to parameter changes. This analysis elucidates how alterations in kinetic parameters affect the behavior of the TP53 network, offering crucial information for understanding its regulatory mechanisms and potential therapeutic interventions.

### 5.4. Frequency Analysis

**Figure 20:**
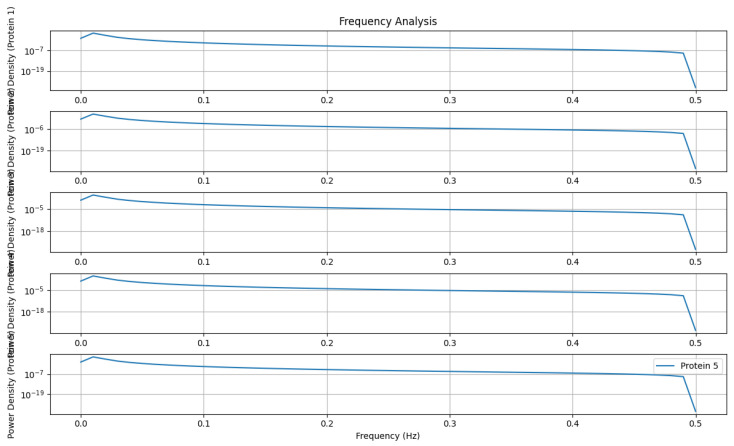
Frequency analysis.

Figure 20 presents a frequency analysis that calculates power spectral density using the Welch method. Subplots display power density spectra for specific proteins, helping identify dominant frequencies in protein concentration signals. This analysis enables the characterization of temporal dynamics within the TP53 network, uncovering oscillatory patterns and regulatory motifs that govern its activity.

## 6. Discussion

The investigation into TP53 overexpression across various cancers has yielded compelling findings regarding its association with overall survival (OS) and disease-free survival (DFS). Elevated TP53 expression consistently correlates with shorter OS and poorer prognoses across multiple cancer types, emphasizing its significance in cancer progression. This observation underscores the critical role of TP53 as a biomarker for predicting patient outcomes and guiding treatment decisions. By elucidating the impact of TP53 expression levels on survival outcomes, our study provides valuable insights into the molecular mechanisms driving cancer progression and highlights the potential clinical implications of targeting TP53 in cancer therapy.

Furthermore, the study delves into detailed analyses of TP53 expression patterns, revealing correlations with different pathological stages, thus shedding light on TP53’s role in driving malignant characteristics. By categorizing tumors based on TP53 expression levels and pathological stages, we uncover the intricate relationship between TP53 dysregulation and cancer progression. This nuanced understanding enhances our ability to stratify patients based on their TP53 status and may facilitate the development of personalized treatment approaches tailored to individual tumor characteristics.

In contrast with existing literature, our study presents a scientifically rigorous and comprehensive analysis of TP53 expression dynamics across diverse cancer types and subtypes [36]. Leveraging the extensive dataset from The Cancer Genome Atlas (TCGA) [37], we meticulously explore the intricate molecular landscape of cancer, aiming to discern unique genetic alterations associated with specific cancer types. This meticulous investigation enhances our understanding of TP53’s multifaceted role in cancer biology by dissecting its involvement in various biological pathways [38].

There is a significant difference in the role of p53 in cancer etiology when comparing our results with those of Chunyan Gao and Fangqi Chen regarding the dynamics of p53 in the DNA damage response. Our research highlights the necessity for focused therapy approaches and highlights the diverse role of increased p53 beyond tumor suppression by indicating that it may unintentionally promote carcinogenesis. On the other hand, Gao and Chen argue that Mdm2 delays induced pulsatile p53 expression and affects DNA damage responses, indicating possible targets for therapy. Despite their differences, both studies highlight the complex function that p53 plays in cancer and call for more research to develop specialized treatments [18,19].

Comparing our results to those reported in the Donehower et al. study [39] reveals that TP53 mutations play a critical role in the biology of cancer. Although the focus of our research is on the contradictory effects of the overexpression of TP53 on carcinogenesis in different forms of cancer, Donehower et al.’s work [39] offers a thorough examination of TP53 mutations and their consequences for patient survival. While Donehower et al.’s work [39] explores the molecular pathways behind TP53 variations across multiple malignancies, our analysis reveals TP53 overexpression as a strong prognostic predictor, notably in prostate adenocarcinoma. Despite these variations, both pieces of research stress how crucial it is to combine various analyses to improve our knowledge of TP53 in cancer and provide specialized treatment approaches. When incorporating knowledge from TP53 expression studies, it is essential to combine dynamics with a thorough assessments of TP53 mutations to inform precision medicine methods for the treatment of cancer.

Significant insights into p53’s function in treatment outcomes are provided by the study examining its prognostic value in chemotherapy response in advanced breast cancer. It also reveals a close relationship between the efficacy of chemotherapy, especially FEC, and p53 status in breast cancers. Interestingly, treatment failure with FEC is highly correlated with positive p53 immunohistochemistry (IHC) and TP53 mutations, emphasizing the critical role that intact p53 function plays in modulating the cytotoxic effects of those drugs that damage DNA. On the other hand, paclitaxel response, which is thought to be independent of p53 function, does not show a correlation with p53 status. However, a correlation between aberrant p53 and paclitaxel response is noted, indicating that tumors may become more sensitive to this chemotherapy regimen if p53 is defective [40]. This observation is consistent with our research about the contradictory function of overexpressed p53 in the etiology of cancer and its possible effects on treatment outcomes. The breast cancer study offers important clinical insights into chemotherapy response based on p53 status, highlighting the variability in chemotherapy efficacy based on tumor p53 status and the potential for customizing treatment strategies to optimize therapeutic outcomes in breast cancer and other malignancies [40]. Our study also sheds light on the broader landscape of p53 dysregulation across various cancer types and its impact on disease-free survival (DFS).

Through our exploration of TP53’s engagement in fundamental biological processes such as cell cycle control and apoptosis [41], we transcend mere correlation analysis. We endeavor to elucidate the intricate regulatory mechanisms orchestrated by TP53, unraveling how its dysregulation exerts influence on the essential cellular functions crucial to cancer development and progression [42].

Our integrative approach goes beyond surface-level analysis, unveiling novel associations between TP53 dysregulation and the pivotal signaling pathways implicated in cancer pathogenesis. By systematically dissecting these molecular interactions, we unveil the intricate crosstalk between TP53 and other molecular constituents, thereby deepening our comprehension of the complex molecular network governing cancer biology.

Our study represents a significant scientific advancement, offering a comprehensive understanding of TP53’s pivotal role in cancer progression. By elucidating the molecular mechanisms underpinning TP53 dysregulation and its intricate connections to key signaling pathways [43], we provide a foundation for the development of precision therapeutic interventions aimed at disrupting these pathways and improving clinical outcomes across diverse cancer types.

However, it is essential to acknowledge the study’s limitations. Our approach heavily relies on bioinformatics tools and databases, potentially introducing biases or inaccuracies into the analysis. While TCGA [44] data offer a wealth of information, it is essential to validate our findings experimentally to ensure their reliability and robustness. Additionally, the scope of the study is confined to the analysis of existing data, overlooking factors such as tumor heterogeneity and patient demographics, which could provide further context to the findings. Future studies should address these limitations by incorporating experimental validation and considering additional clinical and molecular variables.

Moreover, one key limitation arises from our methodology in handling the false discovery rate (FDR). We included data points with *p*-values less than or equal to 0.05 without conducting additional adjustments for multiple comparisons, which could introduce the risk of type I errors, especially considering the multiple comparisons across various datasets. This aspect of our methodological approach could potentially affect the interpretation of our results and the generalizability of the study. We recommend that future research includes more rigorous statistical controls to mitigate this limitation.

Despite these limitations, the implications of our study are significant, extending beyond research to clinical practice and drug development. The insights gained into the complex interplay between TP53 expression and cancer progression can inform tailored treatment strategies based on individual tumor characteristics. Furthermore, the identification of unique genetic alterations associated with specific cancer types opens avenues for targeted therapies and personalized medicine approaches. Ultimately, our study underscores the importance of continued research into TP53 biology for advancing cancer diagnostics and therapeutics, emphasizing the need for multidisciplinary approaches integrating genomic, transcriptomic, and proteomic data to unravel the complexities of TP53 signaling in cancer.

Comparing our findings with available literature provides a broader perspective on the significance of p53 overexpression in tumors and its clinical implications. Our study reinforces the well-established notions of p53 as the “guardian of the genome” [1] and its association with aggressive cancers. Moreover, our comprehensive analysis extends these concepts to a broader array of cancer types, offering insights that can inform therapeutic strategies and emphasizing the need for precision medicine approaches based on TP53 status. By integrating findings from multiple studies, we consolidate existing knowledge and identify areas for future research aimed at elucidating the molecular mechanisms underlying TP53 dysregulation in cancer.

In conclusion, our study leverages TCGA data to investigate TP53 expression patterns, providing robust and consistent findings that echo prior research in the field. The exploration of p53’s pathological stages introduces an innovative dimension to our understanding of its clinical implications, complementing and extending the work of previous scholars. These insights collectively reinforce the significance of TP53 in the landscape of cancer biology and underscore its utility as a prognostic marker with multifaceted roles in cancer progression. By elucidating the molecular mechanisms driving TP53 dysregulation and its implications for cancer prognosis and treatment, our study contributes to the growing body of evidence supporting TP53 as a central player in cancer biology.

## 7. Summary

- TP53, a crucial tumor suppressor gene, regulates cell cycle balance, but elevated expression can promote tumor development.
- Various cancers exhibit heightened TP53 expression, correlating with aggressive behavior and higher recurrence risk.
- Treatment strategies for tumors with TP53 overexpression involve a combination of targeted therapies, chemotherapy, and radiation therapy.
- Analysis of TP53 gene expression across TCGA tumors reveals significant overexpression in 13 out of 27 cancers.
- P53 expression levels categorize tumors into five pathological stages, with higher expression linked to poorer prognosis.
- TP53 manifests in four subtypes: wild-type, mutant, overexpressed, and deleted, each requiring tailored treatment approaches.
- Investigation into TP53 expression across histological and molecular cancer subtypes highlights significant upregulation compared to normal tissues.
- Detailed analysis within specific cancers reveals significant associations between TP53 expression and subtypes, grades, and patient conditions.
- The complexity and heterogeneity of TP53 expression underscores the need for nuanced analyses in cancer research.

## 8. Conclusions

The role of p53 in cancer biology is multifaceted, with both tumor suppressor and oncogenic functions depending on its expression levels and mutation status. Heightened p53 expression is associated with aggressive tumor behavior and poorer prognosis across various cancer types. Tailored treatment strategies involving a combination of therapies are essential for managing tumors with p53 overexpression. Analysis of p53 expression patterns provides valuable insights into tumor classification, prognosis prediction, and treatment selection. However, the complexity and heterogeneity of p53 expression underscore the need for further research to unravel its clinical and biological significance fully. Understanding the intricacies of p53 expression will pave the way for more effective therapeutic interventions and improved outcomes for cancer patients.

## Figures and Tables

**Table 1:** Statistically significant TP53 overexpression based on histological, molecular subtypes, and different patient statuses (only findings with *p*-value < 0.05 are given).

Tumor Type	Histological Subtypes	Molecular Subtypes	Tumor Grade	Other Patient Conditions
ine BRCA	N vs. IDC	$2.774200\times 10^{-4}$		
			N vs. Luminal	$6.559900\times 10^{-3}$
			N vs. Post-Menopause	$7.136300\times 10^{-3}$
	N vs. Mucinous	$2.781400\times 10^{-1}$		
			Pre-Menopause vs. Peri-Menopause	$1.829550\times 10^{-1}$
	N vs. Medullary	$1.235400\times 10^{-1}$		
			Pre-Menopause vs. Post-Menopause	$1.253980\times 10^{-1}$
	IDC vs. ILC	$1.340450\times 10^{-3}$		
	IDC vs. Mucinous	$5.196900\times 10^{-3}$		
	IDC vs. Medullary	$2.930100\times 10^{-2}$		
	ILC vs. Medullary	$1.431390\times 10^{-1}$		
	Mixed vs. Mucinous	$6.823700\times 10^{-2}$		
	Other vs. Mucinous	$1.254160\times 10^{-2}$		
ine OV			Grade 2 vs. Grade 3	$6.921400\times 10^{-1}$
ine TGCT	Seminoma vs. Non Seminoma	$4.0364\times 10^{-5}$		
ine KIRC	N vs. ccA subtype	$4.99522645469597\times 10^{-12}$		
			N vs. Grade 1	$5.2377\times 10^{-4}$
	N vs. ccB subtype	$1.98985272703567\times 10^{-12}$	N vs. Grade 2	$2.11941575400942\times 10^{-13}$
			N vs. Grade 3	$3.15403259065761\times 10^{-12}$
			N vs. Grade 4	$1.01290087428652\times 10^{-11}$
			Grade 2 vs. Grade 4	$3.5742\times 10^{-2}$
			Grade 3 vs. Grade 4	$3.0098\times 10^{-2}$
ine KIRP	N vs. Type1 PRCC	$1.62481139653892\times 10^{-12}$		
	N vs. Type2 PRCC	$5.04439999993167\times 10^{-7}$		
	N vs. Other	$3.9384\times 10^{-2}$		
	N vs. Unclassified PRCC	$3.22909999961318\times 10^{-7}$		
	Type1 PRCC vs. Type2 PRCC	$2.21339999999515\times 10^{-5}$		
	Type1 PRCC vs. KIRP CIMP	$3.8202\times 10^{-2}$		
	Type2 PRCC vs. KIRP CIMP	$8.6439\times 10^{-3}$		
	Type2 PRCC vs. Unclassified PRCC	$3.6119\times 10^{-2}$		
	KIRP CIMP vs. Unclassified PRCC	$2.9879\times 10^{-2}$		
ine PRAD	N vs. ERG fusion	$1.53808999999727\times 10^{-5}$		
	N vs. Gleason score 6	$1.89542\times 10^{-3}$		
	N vs. Gleason score 7	$2.7754\times 10^{-4}$		
	N vs. Gleason score 8	$5.2036\times 10^{-2}$		
	Gleason score 7 vs. Gleason score 9	$1.16399\times 10^{-3}$		
ine BLCA	N vs. Neuronal	$1.64178\times 10^{-2}$		
	N vs. NonPapillary tumors	$3.8151\times 10^{-3}$		
	N vs. Basal squamous	$4.7461\times 10^{-2}$		
	N vs. Mixed	<$10^{-6}$		
	N vs. TNBC	<$10^{-12}$		
	N vs. Post-Menopause	<$10^{-12}$		
	N vs. Luminal	$4.4781\times 10^{-3}$		
	N vs. Luminal_Papillary	$3.8442\times 10^{-5}$		
ine COAD	N vs. Adenocarcinoma	$1.62447832963153\times 10^{-12}$		
	N vs. Mucinous Adenocarcinoma	$3.42909999950791\times 10^{-7}$		
ine ESCA	N vs. Adenocarcinoma	$7.25219884145645\times 10^{-11}$		
			N vs. Grade 1	$4.1368\times 10^{-3}$
			N vs. Grade 2	$1.42999945218492\times 10^{-10}$
			N vs. Grade 3	$4.56420000016777\times 10^{-7}$
			N vs. Grade 4	$1.21014309684142\times 10^{-14}$
			Grade 1 vs. Grade 3	$1.4914000034949\times 10^{-9}$
			Grade 4 vs. Grade 5	$2.964399\times 10^{-1}$
ine HNSC			N vs. Grade 1	$4.7443\times 10^{-2}$
			Normal vs. HPV+ve	$6.43040065639866\times 10^{-11}$
			N vs. Grade 2	$2.3995\times 10^{-4}$
			N vs. Grade 3	$4.56420000016777\times 10^{-7}$
			N vs. Grade 4	$1.21014309684142\times 10^{-14}$
			Grade 1 vs. Grade 3	$1.4914000034949\times 10^{-9}$
			Grade 4 vs. Grade 5	$2.964399\times 10^{-1}$
ine LIHC			Grade 1 vs. Grade 3	$5.7901\times 10^{-4}$
			Grade 2 vs. Grade 3	$1.15593\times 10^{-2}$
ine READ	N vs. Adenocarcinoma	$9.20689999794888\times 10^{-8}$		
	N vs. Mucinous-adenocarcinoma	$4.3804\times 10^{-3}$		
ine PAAD			N vs. Non Drinker	$3.7197\times 10^{-2}$
			N vs. Daily Drinker	$3.3847\times 10^{-3}$
			N vs. Occasional Drinker	$3.8601\times 10^{-2}$
			N vs. Social Drinker	$4.1244\times 10^{-3}$
ine LGG	Astrocytoma vs. Oligoastrocytoma	$1.91464\times 10^{-1}$ (*p*-value = 0.191464)	Grade 2 vs. Grade 3	$6.68579969165251\times 10^{-10}$
	Astrocytoma vs. Oligodendroglioma	$8.841000\times 10^{-1}$ (*p*-value = 0.8841)		
	Oligoastrocytoma vs. Oligodendroglioma	$2.020400\times 10^{-1}$ (*p*-value = 0.20204)		
ine STAD	N vs. Adenocarcinoma(NOS)	<$1\times 10^{-12}$		
			N vs. Grade 1	$7.2362\times 10^{-4}$
	N vs. Tumors (with H.pylori infection)	$1.11651\times 10^{-3}$		
	N vs. Adenocarcinoma(Diffuse)	$1.26749999385112\times 10^{-9}$		
			N vs. Grade 2	<$1\times 10^{-12}$
			N vs. Tumors (without H.pylori infection)	<$1\times 10^{-12}$
	N vs. IntestinalAdenocarcinoma(NOS)	$1.44750000874438\times 10^{-9}$		
	N vs. IntestinalAdenocarcinoma(Tubular)	$3.25794946576252\times 10^{-12}$		
	N vs. IntestinalAdenocarcinoma(Mucinous)	$2.8434\times 10^{-4}$		
	Normal vs. IntestinalAdenocarcinoma(Papillary)	$2.2975\times 10^{-2}$		
			Grade 1 vs. Grade 3	0
ine LUAD	N vs. NOS	<$1\times 10^{-12}$		
	Normal vs. Mixed	$2.30981900273264\times 10^{-12}$		
	N vs. ClearCell	$5.8418\times 10^{-4}$		
	N vs. LBC-NonMucinous	$2.2229\times 10^{-3}$		
	N vs. Papillary	$1.46265\times 10^{-3}$		
	N vs. Mucinous	$4.24039999999959\times 10^{-5}$		
	N vs. Acinar	$5.4213\times 10^{-4}$		
ine LUSC	N vs. NOS	<$1\times 10^{-12}$		
	Normal vs. Basaloid	$3.1045\times 10^{-2}$		
ine UCEC	N vs. Endometrioid	$4.05908640033203\times 10^{-12}$		
			N vs. Pre-Menopause	$1.60299999940605\times 10^{-8}$
			N vs. Peri-Menopause	$5.50919999999788\times 10^{-5}$
			N vs. Post-Menopause	$1.32620026072061\times 10^{-10}$
	Endometrioid vs. Mixed serious	$1.23953\times 10^{-2}$		
ine THYM	Type A vs. Type AB	$2.5563\times 10^{-4}$		
	Type A vs. Type B1	$5.6701\times 10^{-10}$		
	Type A vs. Type B2	$3.7402\times 10^{-9}$		
	Type A vs. Type B2|B3	$7.8849\times 10^{-3}$		
	Type A vs. Other	$5.67240000031188\times 10^{-4}$		
ine UCS			N vs. Serous-like endometrial carcinoma	$2.6023\times 10^{-2}$
	N vs. Endometrioid	$3.46550000000042\times 10^{-10}$		
	N vs. Carcinosarcoma	$6.9539\times 10^{-4}$		
	N vs. Serous-like ovarian carcinoma	$4.7403\times 10^{-3}$		
	Serous-like endometrial carcinoma vs. Endometrioid	$2.0281\times 10^{-2}$		
	Serous-like endometrial carcinoma vs. Serous-like ovarian carcinoma	$3.36269999999943\times 10^{-5}$		

**Table 2:** Unique Genes in Each Cancer Type.

Cancer Type	Unique Genes
BRCA	BRCA1, PALB2, ELAC2, EZH2, FOXM1, AURKA, AURKB, BUB1B
COAD	BRCA1, PALB2, ELAC2, EZH2, FOXM1, AURKA, AURKB, BUB1B
KICH	ELAC2, BUB3, CHEK2
KIRC	ELAC2, EZH2
KIRP	EZH2, BRCA1, ATM, AURKB, BUB3
LIHC	BUB1, ELAC2, PLK1, AURKB
LUSC	ELAC2
DLBC	BRCA1, PALB2, ELAC2, EZH2, FOXM1, AURKA, AURKB, BUB1B
MESO	FOXM1, BRCA1, MYBL2, TTK, AURKB
PAAD	BUB1, BRCA1, CHEK2, E2F1, EZH2, MYBL2, TTK, AURKA, AURKB
PCPG	ELAC2
PRAD	ELAC2
READ	ELAC2, AURKB
TGCT	AURKB
THCA	BRCA1, BUB1B, EZH2, PALB2
THYM	BRCA1, PALB2, AURKA
UCEC	ATR, BRCA1, BRCA2, ELAC1, ELAC2

**Table 3:** Expression based on TP53 in Mutation Comparison.

Type of Cancer	Comparison	Statistical Significance
ine BLCA	Normal vs. TP53-Mutant	$5.243300\times 10^{-3}$
	Normal vs. TP53-NonMutant	$1.141710\times 10^{-3}$
	TP53-Mutant vs. TP53-NonMutant	$5.716600\times 10^{-1}$
ine BRCA	Normal vs. TP53-Mutant	$9.097500\times 10^{-3}$
	Normal vs. TP53-NonMutant	$5.359400\times 10^{-4}$
	TP53-Mutant vs. TP53-NonMutant	$1.332590\times 10^{-1}$
ine COAD	Normal vs. TP53-Mutant	$3.77475828372553\times 10^{-15}$
	Normal vs. TP53-NonMutant	$1.62447832963153\times 10^{-12}$
	TP53-Mutant vs. TP53-NonMutant	$3.74690000000122\times 10^{-5}$
ine ACC	TP53-Mutant vs. TP53-NonMutant	$8.257800\times 10^{-1}$
ine LGG	TP53-Mutant vs. TP53-NonMutant	$6.669000\times 10^{-1}$
ine BRCA based on MYC	MYC-amplification(+) vs. MYC-amplification(−)	$8.445400\times 10^{-1}$
BRCA based on CCND1	CCND1-amplification(+) vs. CCND1-amplification(−)	$5.233700\times 10^{-2}$
BRCA based on ERBB2	ERBB2-amplification(+) vs. ERBB2-amplification(−)	$9.315000\times 10^{-1}$
ine COAD	Normal vs. TP53-Mutant	$3.77475828372553\times 10^{-15}$
	Normal vs. TP53-NonMutant	$1.62447832963153\times 10^{-12}$
	TP53-Mutant vs. TP53-NonMutant	$3.74690000000122\times 10^{-5}$
ine ESCA	Normal vs. TP53-Mutant	$1.81366033302766\times 10^{-12}$
	Normal vs. TP53-NonMutant	$4.35860000003174\times 10^{-6}$
	TP53-Mutant vs. TP53-NonMutant	$1.304420\times 10^{-2}$
ine GBM	Normal vs. TP53-Mutant	$1.63613567139009\times 10^{-12}$
	Normal vs. TP53-NonMutant	<$1\times 10^{-12}$
	TP53-Mutant vs. TP53-NonMutant	$8.685000\times 10^{-1}$
ine HNSC	Normal vs. TP53-Mutant	$1.207930\times 10^{-2}$
	Normal vs. TP53-NonMutant	$4.03779998325859\times 10^{-9}$
	TP53-Mutant vs. TP53-NonMutant	$1.53136999999637\times 10^{-5}$
KICH	Normal vs. TP53 Mutant	$3.990400\times 10^{-4}$
	Normal vs. TP53 Non-Mutant	$7.3109999998433\times 10^{-7}$
	TP53 Mutant vs. TP53 Non-Mutant	$7.653200\times 10^{-1}$
ine LIHC	Normal vs. TP53 Mutant	$1.98754999999329\times 10^{-5}$
	Normal vs. TP53 Non-Mutant	<$1\times 10^{-12}$
	TP53 Mutant vs. TP53 Non-Mutant	$5.692600\times 10^{-1}$
ine LUAD	Normal vs. TP53 Mutant	$2.18425277864753\times 10^{-12}$
	Normal vs. TP53 Non-Mutant	$1.11022302462516\times 10^{-16}$
	TP53 Mutant vs. TP53 Non-Mutant	$6.271800\times 10^{-2}$
LUSC	Normal vs. TP53 Mutant	<$1\times 10^{-12}$
	Normal vs. TP53 Non-Mutant	$5.06979999981283\times 10^{-7}$
	TP53 Mutant vs TP53 Non-Mutant	$5.819700\times 10^{-2}$
ine DLBC	TP53 Mutant vs. TP53 Non-Mutant	$8.984400\times 10^{-2}$
ine MESO	TP53 Mutant vs. TP53 Non-Mutant	$5.87539999999942\times 10^{-5}$
ine OV	TP53 Mutant vs. TP53 Non-Mutant	$8.243400\times 10^{-1}$
PAAD	Normal vs. TP53 Mutant	$4.129700\times 10^{-2}$
	Normal vs. TP53 Non-Mutant	$7.754100\times 10^{-2}$
	TP53 Mutant vs. TP53 Non-Mutant	$6.186000\times 10^{-1}$
ine PRAD	Normal vs. TP53 Mutant	$8.082600\times 10^{-1}$
	Normal vs. TP53 Non-Mutant	$1.739050\times 10^{-3}$
	TP53 Mutant vs. TP53 Non-Mutant	$5.738700\times 10^{-3}$
ine READ	Normal vs. TP53 Mutant	$5.56630297410265\times 10^{-11}$
	Normal vs. TP53 Non-Mutant	$4.77629999995344\times 10^{-8}$
	TP53 Mutant vs. TP53 Non-Mutant	$7.376800\times 10^{-1}$
ine SARC	Normal vs. TP53 Mutant	$3.776000\times 10^{-1}$
	Normal vs. TP53 Non-Mutant	$4.223600\times 10^{-1}$
	TP53 Mutant vs. TP53 Non-Mutant	$8.565800\times 10^{-1}$
ine SKCM	Normal vs. TP53 Mutant	N/A
	Normal vs. TP53 Non-Mutant	N/A
	TP53 Mutant vs. TP53 Non-Mutant	$2.50359732945071\times 10^{-11}$
ine STAD	Normal vs. TP53 Mutant	$1.62436730732907\times 10^{-12}$
	Normal vs. TP53 Non-Mutant	<$1\times 10^{-12}$
	TP53 Mutant vs. TP53 Non-Mutant	$9.5924000000025\times 10^{-5}$
ine UCS	TP53 Mutant vs. TP53 Non-Mutant	$7.412600\times 10^{-1}$
ine UCES	Normal vs. TP53 Mutant	$1.74265999999257\times 10^{-5}$
	Normal vs. TP53 Non-Mutant	$2.37354580434612\times 10^{-12}$
	TP53 Mutant vs. TP53 Non-Mutant	$1.796110\times 10^{-3}$

**Table 4:** Biological Pathways Related to TP53.

KEGG Number	Cellular Process	Protein
ine hsa04115	p53 signaling pathway	p53 apoptosis effector related to PMP22 (PERP)
ine hsa01522	Endocrine resistance	cytochrome P450 family 2 subfamily D member 6
ine hsa01524	Platinum drug resistance	ERCC1 MLH1 MSH2 GSTP1
hsa:9537	Not assigned	Tumor protein p53 inducible protein 11 (TP53I11, PIG11)
ine hsa:94241	Not Included in Pathway or Brite	Tumor protein p53 inducible nuclear protein 1 (TP53INP1, SIP, TP53DINP1, TP53INP1A, TP53INP1B, Teap, p53DINP1)
ine hsa04010	MAPK signaling pathway	MAPK signaling
ine hsa04014	Ras signaling pathway, Ras signaling	RAS, RTK, GRB2, SOS, RAS
		RAF, MEK, ERK
hsa04110	Cell cycle	BUB1B, BUBR1, MAD3L, BUB3, BUB3L, hBUB3, BUB1B, BUB1beta, BUBR1, Bub1A, MAD3L, MVA1, SSK1, hBUBR1
ine hsa04115	p53 signaling pathway	Serine/threonine kinases: AGC group Serine/threonine kinases: CAMK group Serine/threonine kinases: CK1 group Serine/threonine kinases: CMGC group Serine/threonine kinases: STE group Serine/threonine kinases: TKL group Serine/threonine kinases: Other Receptor serine/threonine kinases (RSTK): TKL group Receptor tyrosine kinases (RTK) Non-receptor tyrosine kinases Histidine kinases
ine hsa04151	PI3K-Akt signaling pathway GF-EGFR-PI3K signaling pathway	EGF; epidermal growth factor
ine		EGFR; epidermal growth factor receptor
		PIK3CA; phosphatidylinositol-4,5-bisphosphate 3-kinase catalytic subunit alpha
		PIK3CB; phosphatidylinositol-4,5-bisphosphate 3-kinase catalytic subunit beta
		PIK3CD; phosphatidylinositol-4,5-bisphosphate 3-kinase catalytic subunit delta
		AKT1; AKT serine/threonine kinase 1
		AKT2; AKT serine/threonine kinase 2
		AKT3; AKT serine/threonine kinase 3
		BAD; BCL2 associated agonist of cell death
hsa04210	Apoptosis Extrinsic apoptotic pathway	TNF; tumor necrosis factor
		TNFRSF1A; TNF receptor superfamily member 1A
		RIPK1; receptor interacting serine/threonine kinase 1
		TRADD; TNFRSF1A associated via death domain
		TRAF2; TNF receptor associated factor 2
		TRAF5; TNF receptor associated factor 5
		FADD; Fas associated via death domain
		CASP8; caspase 8
		CASP3; caspase 3
		CASP7; caspase 7
ine hsa05200	Pathways in cancer EGF-EGFR-RAS-ERK signaling pathway	EGF; epidermal growth factor
		EGFR; epidermal growth factor receptor
		GRB2; growth factor receptor bound protein 2
		SOS1; SOS Ras/Rac guanine nucleotide exchange factor 1
		SOS2; SOS Ras/Rho guanine nucleotide exchange factor 2
		HRAS; HRas proto-oncogene, GTPase
		KRAS; KRAS proto-oncogene, GTPase
		NRAS; NRAS proto-oncogene, GTPase
		ARAF; A-Raf proto-oncogene, serine/threonine kinase
		BRAF; B-Raf proto-oncogene, serine/threonine kinase
		RAF1; Raf-1 proto-oncogene, serine/threonine kinase
		MAP2K1; mitogen-activated protein kinase kinase 1
		MAP2K2; mitogen-activated protein kinase kinase 2
		MAPK1; mitogen-activated protein kinase 1
		MAPK3; mitogen-activated protein kinase 3
		CCND1; cyclin D1
ine hsa04216	Ferroptosis Glutathione biosynthesis	Cys+Glu – (GCLC+GCLM) » GSS -> GSH – GPX -> GSSG
ine hsa04218	Cellular senescence	TGFβ, TGFBR, SMAD, CDK, CCND, RB, E2F, PIK3CA, FOX, MHC, Ras, AKT, TSC, mTOR, PTEN, SIRT, HLA, KIR, MYB, RB, MYC, MYC, MDM2, TP53, βTrCP, HIPK
ine		PP1,RAF MAP2K1, ETS1 GADD45A, GADD45, CDK1, MRE11, ATM, RAD9A, RAD1
		ATR, CDC25A SQSTM1, GATA4 TRAF3IP2, NFKB1 IL1A IGFBP3
ine hsa04120	Ubiquitin mediated proteolysis	BRCA1, CDC20, UBE2C, UBE2S
ine hsa04068	FoxO signaling pathway	ATM, CCNB1, CCNB2, CDKN1B, PLK1, PLK4
ine hsa03030	DNA replication	FEN1, RNASEH2A
ine hsa03440	Homologous recombination	ATM, BRCA1, BRCA2, RAD54L
ine hsa01522	Endocrine resistance	E2F1, TP53
ine hsa03460	Fanconi anemia pathway	ATR, BRCA1, BRCA2, UBE2T
ine hsa05200	Pathways in cancer	BRCA2, CKS2, E2F1, TP53
ine hsa04151	PI3K-Akt signaling pathway	BRCA1, TP53
ine hsa05202	Transcriptional misregulation in cancer	ATM, TP53
ine hsa03410	Base excision repair	FEN1
ine hsa05222	Small cell lung cancer	CKS2, E2F1, TP53
ine hsa05215	Prostate cancer	E2F1, TP53
ine hsa05226	Gastric cancer	E2F1, TP53
ine hsa05220	Chronic myeloid leukemia	E2F1, TP53
ine hsa05219	Bladder cancer	E2F1, TP53
ine hsa05214	Glioma	E2F1, TP53
ine hsa05218	Melanoma	E2F1, TP53
ine hsa05212	Pancreatic cancer	BRCA2, E2F1, TP53
ine hsa05224	Breast cancer	BRCA1, BRCA2, E2F1, TP53
ine hsa05223	Non-small cell lung cancer	E2F1, TP53
ine hsa04151 hsa04919	Ovarian Cancer	BRCA1 BRCA2 MSH2 MLH1 ERBB2 K-ras AKT2 PIK3CA c-MYC p53 CTNNB1 PRKN OPCML AKT1 CDH1//

## Data Availability

The datasets generated and analyzed during the current study are available from the corresponding author upon reasonable request.

## Supplementary Materials

The following supporting information can be downloaded at: https://www.mdpi.com/article/10.3390/genes15050577/s1, In this study, supplementary information including figures and tables were provided to enhance the understanding of the experimental procedures and results. Supplementary File S1 depicts the complete explanation of Table 1, and Supplementary File S2: OS and Supplementary File S3: DFS provide the OS and DFS details of all the cancers.
