# Supplementary material for: Comprehensive Bioinformatic Investigation of TP53 Dysregulation in Diverse Cancer Landscapes
Source: Genes (Basel). 2024 Apr 30;15(5):577. doi: 10.3390/genes15050577 (PMC11121236; doi:10.3390/genes15050577)
Supplement: Supplementary file 1 [file genes-15-00577-s001.zip › genes-2922551-supplementary file S1.pdf]

The provided table presents statistically significant TP53 overexpression findings based on histological subtypes, molecular subtypes, tumor grades, and other patient conditions across various cancer types. Here's an explanation of each component and its role in cancer:

1. Tumor Type: This column specifies the type of cancer being studied.

2. Histological Subtypes: Histological subtypes refer to the different morphological patterns observed in cancer cells. For example, in breast cancer (BRCA), different subtypes such as infiltrating ductal carcinoma (IDC), mucinous, and medullary carcinoma show varying levels of TP53 overexpression compared to normal tissue. These differences in TP53 expression may reflect distinct biological behaviors and clinical outcomes associated with each histological subtype.

3. Molecular Subtypes: Molecular subtypes categorize cancers based on specific genetic or molecular alterations. In some cancers like kidney renal papillary cell carcinoma (KIRP), different molecular subtypes exhibit varying levels of TP53 expression. This suggests that TP53 dysregulation may be linked to specific molecular pathways driving tumor development and progression.

4. Tumor Grade: Tumor grade indicates the degree of abnormality observed in cancer cells and is often associated with aggressiveness and prognosis. For instance, in head and neck squamous cell carcinoma (HNSC), different tumor grades show significant differences in TP53 expression levels. This implies that TP53 dysregulation may contribute to the histological changes associated with tumor progression and differentiation.

5. Other Patient Conditions: This category includes various patient-related factors such as menopausal status, drinking habits, or specific genetic alterations. For example, in prostate adenocarcinoma (PRAD), TP53 expression levels vary based on factors like the presence of ERG fusion or Gleason score. These findings suggest that TP53 dysregulation may interact with other molecular or clinical variables to influence cancer development and response to treatment.

Overall, the findings presented in the table highlight the complex interplay between TP53 expression and various biological and clinical factors in different cancer types. Understanding

these relationships is crucial for elucidating the role of TP53 in cancer pathogenesis and identifying potential therapeutic targets for precision medicine approaches.

Certainly! Let's integrate evidence from the provided table into the descriptions for each cancer type:

1. BRCA (Breast Cancer):

- Role of TP53: In BRCA tumors, specifically in infiltrating ductal carcinoma (IDC), there is a statistically significant overexpression of TP53 compared to normal tissue. This finding, supported by molecular subtypes and patient conditions, suggests a potential role for TP53 in promoting breast cancer progression and may influence treatment responses.

2. COAD (Colon Adenocarcinoma):

- Role of TP53: COAD analysis reveals significant disparities in TP53 expression between adenocarcinoma and normal tissue. This suggests a potential involvement of TP53 in the development and progression of colon adenocarcinoma, indicating its relevance as a biomarker or therapeutic target.

3. ESCA (Esophageal Carcinoma):

- Role of TP53: Although specific histological subtypes are not provided in the table, the associations between TP53 expression and various subtypes, grades, and other conditions in ESCA underscore its involvement in esophageal carcinoma. This suggests that TP53 may play a multifaceted role in esophageal cancer biology, influencing tumor behavior and treatment responses.

4. HNSC (Head and Neck Squamous Cell Carcinoma):

- Role of TP53: Insights into TP53 expression patterns across diverse conditions in HNSC enhance our understanding of its role in head and neck cancer progression. This suggests that TP53 alterations may contribute to the development and aggressiveness of head and neck squamous cell carcinoma, potentially impacting patient outcomes and therapeutic strategies.

5. KIRC (Kidney Renal Clear Cell Carcinoma):

- **Role of TP53**: In KIRC, TP53 shows consistent elevation across all tumor grades, with emphasis on specific molecular subtypes. This indicates that TP53 dysregulation may be a common feature in kidney renal clear cell carcinoma, potentially driving tumor development and progression.

6. KIRP (Kidney Renal Papillary Cell Carcinoma):

- Role of TP53: Differences in TP53 expression across different histological and molecular subtypes, as well as tumor grades in KIRP, suggest its involvement in the heterogeneous nature of kidney renal papillary cell carcinoma. This implies that TP53 alterations may contribute to the diverse clinical behavior observed in this cancer type.

7. LIHC (Liver Hepatocellular Carcinoma):

- Role of TP53: Compelling associations between TP53 expression and distinct tumor grades in LIHC highlight its clinical relevance in liver hepatocellular carcinoma progression and prognosis. This suggests that TP53 dysregulation may serve as a prognostic indicator and potential therapeutic target in LIHC.

8. READ (Rectum Adenocarcinoma):

- Role of TP53: Accentuation of substantial differences in TP53 expression across various histological subtypes in READ offers insights for tailored treatment strategies. This implies that TP53 alterations may influence the biological behavior of rectum adenocarcinoma subtypes, potentially impacting treatment response and patient outcomes.

9. PAAD (Pancreatic Adenocarcinoma):

- Role of TP53: Elucidation of significant associations between TP53 expression and various drinking habits in PAAD suggests implications for personalized patient management. This indicates that TP53 dysregulation may interact with lifestyle factors to influence pancreatic adenocarcinoma development and progression.

10. LGG (Brain Lower Grade Glioma):

- Role of TP53: Significant differences in TP53 expression across diverse histological subtypes and tumor grades in LGG highlight its multifaceted role in glioma biology. This implies that TP53 alterations may contribute to the molecular heterogeneity observed in brain lower grade gliomas, potentially influencing tumor behavior and treatment responses.

11. LUAD (Lung Adenocarcinoma):

- Role of TP53: Insights into TP53 expression patterns amidst different histological subtypes, grades, and other patient conditions in LUAD offer critical knowledge for precision medicine approaches. This suggests that TP53 dysregulation may be a key determinant of lung adenocarcinoma subtypes and may influence patient outcomes and therapeutic responses.

12. LUSC (Lung Squamous Cell Carcinoma):

- Role of TP53: Unraveling significant associations between TP53 expression and various histological subtypes in LUSC enriches our understanding of its involvement in lung squamous cell carcinoma progression. This implies that TP53 alterations may contribute to the diverse clinical behavior observed in lung squamous cell carcinoma, potentially impacting treatment strategies and patient outcomes.

13. OV (Ovarian Serous Cystadenocarcinoma):

- Role of TP53: Marked differences in TP53 expression across different tumor grades in OV provide key insights into its role in ovarian cancer development and prognosis. This suggests that TP53 dysregulation may be a critical factor in determining the aggressiveness of ovarian serous cystadenocarcinoma and could serve as a prognostic marker for patient stratification.

14. PRAD (Prostate Adenocarcinoma):

- Role of TP53: Underscoring significant associations between TP53 expression, Gleason score, and molecular subtypes in PRAD offers valuable prognostic markers. This indicates that TP53 dysregulation may play a pivotal role in prostate adenocarcinoma progression and could aid in risk stratification and treatment decision-making.

15. STAD (Stomach Adenocarcinoma):

- Role of TP53: Offering intricate insights into diverse aspects of the disease, STAD analysis reveals significant associations between TP53 expression and various subtypes, grades, other patient conditions, and tumor stages. This illuminates the complex landscape of stomach adenocarcinoma biology and suggests that TP53 dysregulation may contribute to the heterogeneous clinical behavior observed in this malignancy.

16. TGCT (Testicular Germ Cell Tumors):

- Role of TP53: Unveiling substantial differences in TP53 expression between seminoma and non-seminoma subtypes in TGCT sheds light on its role in testicular germ cell tumor biology. This suggests that TP53 dysregulation may be implicated in the distinct molecular pathways underlying seminoma and non-seminoma development, potentially influencing tumor behavior and treatment responses.

17. THYM (Thymoma):

- Role of TP53: Elucidating profound distinctions between different types and subtypes of THYM provides invaluable insights for diagnostic and therapeutic strategies. This suggests that TP53 dysregulation may contribute to the molecular heterogeneity observed in thymoma, potentially impacting disease management and treatment outcomes.

18. UCEC (Uterine Corpus Endometrial Carcinoma):

- Role of TP53: Unraveling exceptional significance in various comparisons in UCEC indicates substantial differences with profound clinical implications. This implies that TP53 dysregulation may be a critical driver of uterine corpus endometrial carcinoma pathogenesis and could serve as a potential therapeutic target or prognostic marker.

19. UCS (Uterine Carcinosarcoma):

- Role of TP53: Providing illuminating insights into TP53 expression patterns across diverse histological subtypes in UCS enriches our understanding of its involvement in uterine carcinosarcoma progression. This suggests that TP53 dysregulation may contribute to the complex biology of uterine carcinosarcoma, potentially influencing treatment strategies and patient outcomes.

These findings underscore the diverse and multifaceted role of TP53 dysregulation across different cancer types, highlighting its potential as a diagnostic, prognostic, and therapeutic biomarker in cancer management.
