# Supplementary material for: Comprehensive Bioinformatic Investigation of TP53 Dysregulation in Diverse Cancer Landscapes
Source: Genes (Basel). 2024 Apr 30;15(5):577. doi: 10.3390/genes15050577 (PMC11121236; doi:10.3390/genes15050577)
Supplement: Supplementary file 1 [file genes-15-00577-s001.zip › genes-2922551-supplementary file S2-OS.pdf]

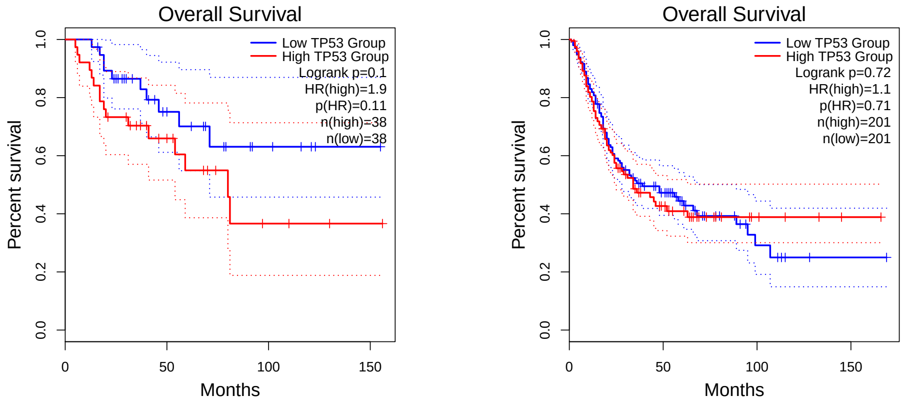

(a) Overall Survival of ACC (b) Overall Survival of BLCA

Figure S1: Overall Survival

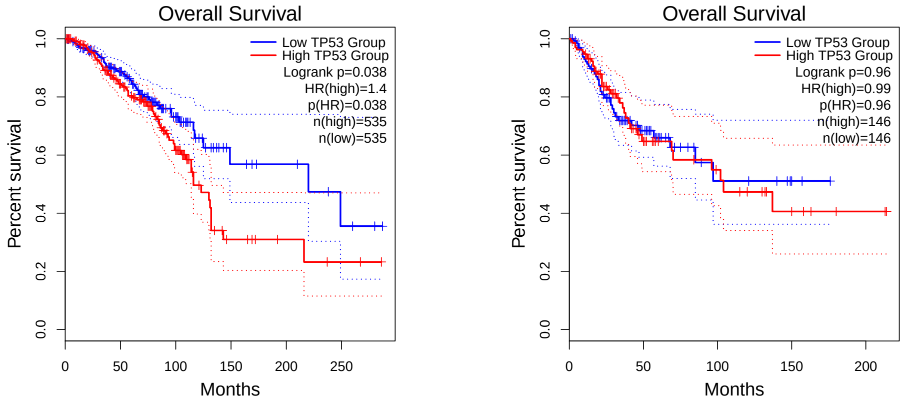

(a) Overall Survival of BRCA (b) Overall Survival of CESC

Figure S2: OVERALL SURVIVLAL

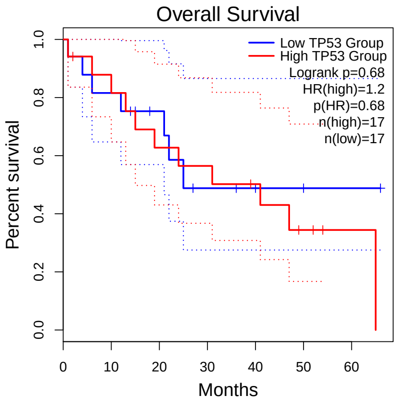

(a) Overall Survival of CHOL

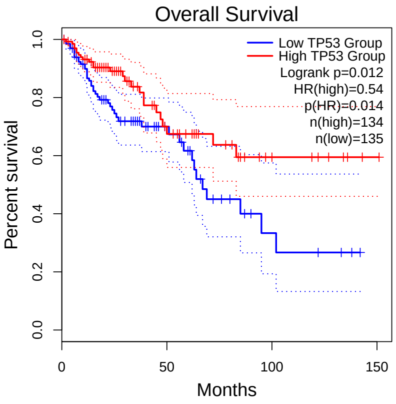

(b) Overall Survival of COAD

Figure S3: Overall Survival

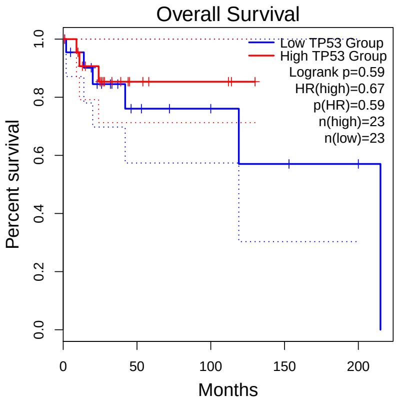

(a) Overall Survival of DLBC

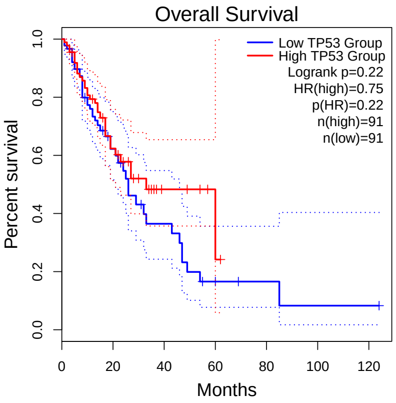

(b) Overall Survival of ESCA

Figure S4: Overall Survival

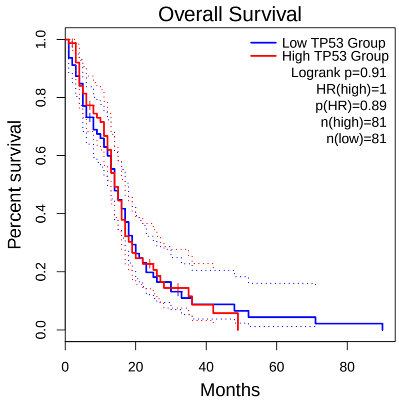

(a) Overall Survival of GBM

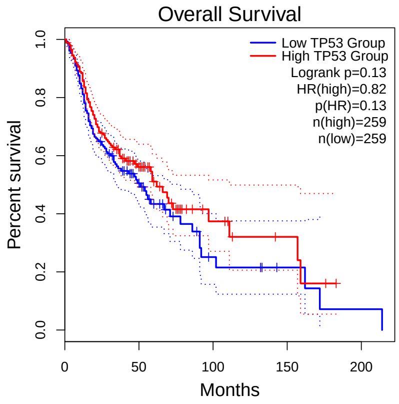

(b) Overall Survival of HNSC

Figure S5: Overall Survival

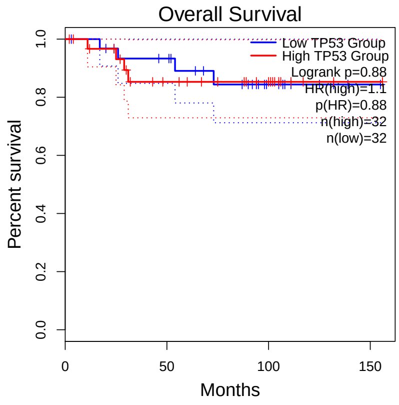

(a) Overall Survival of KICH

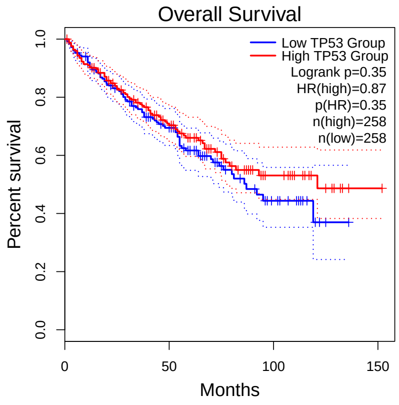

(b) Overall Survival of KIRC

Figure S6: Overall Survival

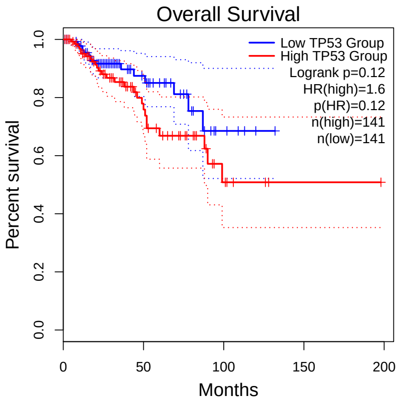

(a) Overall Survival of KIRP

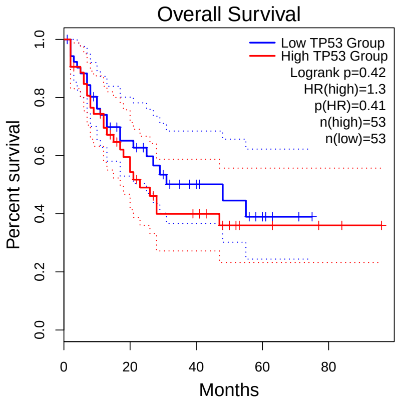

(b) Overall Survival of LAML

Figure S7: Overall Survival

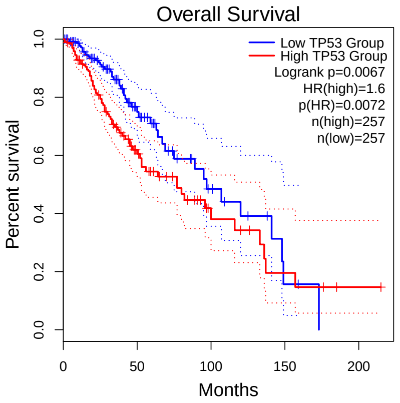

(a) Overall Survival of LGG

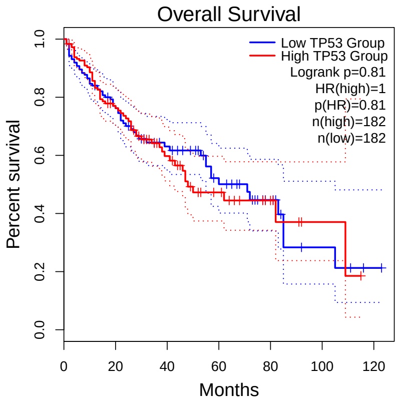

(b) Overall Survival of LIHC

Figure S8: Overall Survival

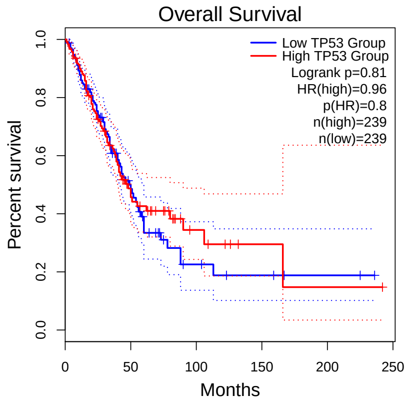

(a) Overall Survival of LUAD

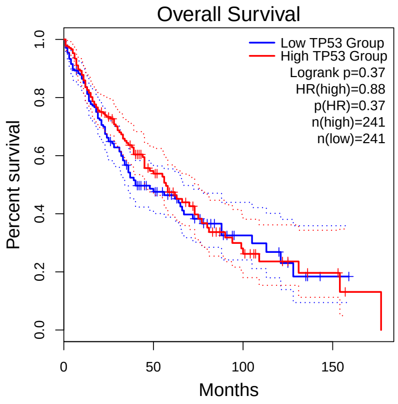

(b) Overall Survival of LUSC

Figure S9: Overall Survival

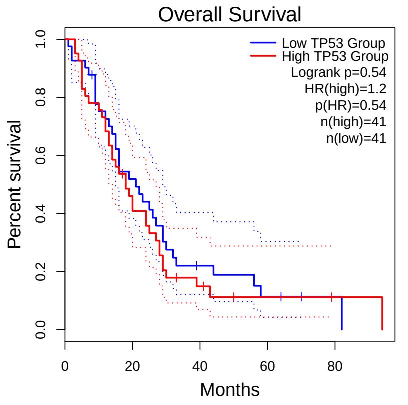

(a) Overall Survival of MESO

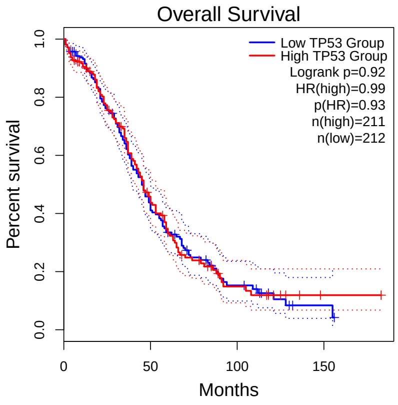

(b) Overall Survival of OV

Figure S10: Overall Caption for both Images

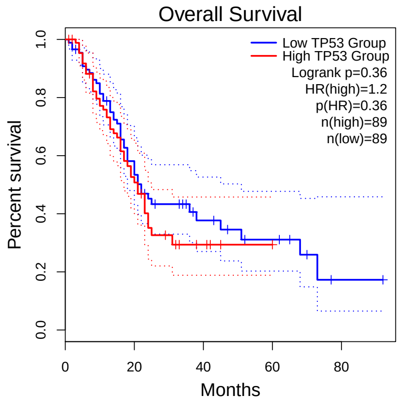

(a) Overall Survival of PAAD

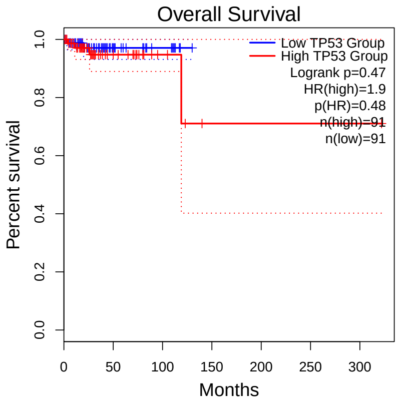

(b) Overall Survival of PCPG

Figure S11: Overall Survival

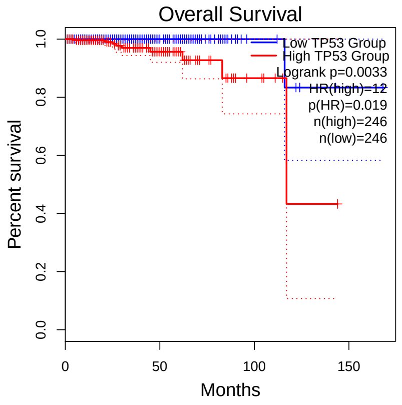

(a) Overall Survival of PRAD

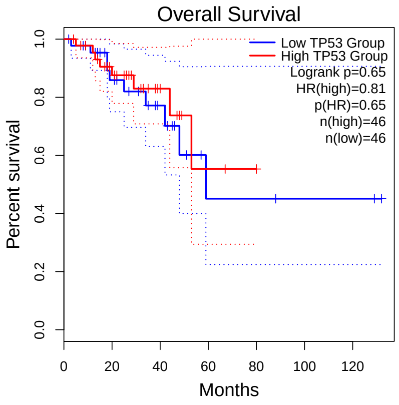

(b) Overall Survival of READ

Figure S12: Overall Survival

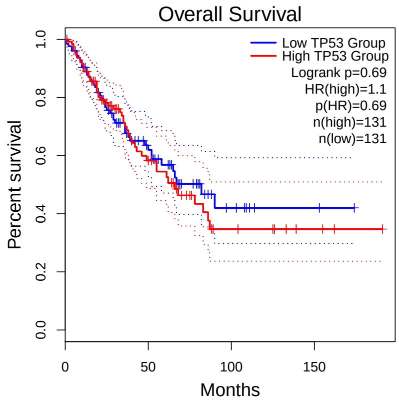

(a) Overall Survival of SARC

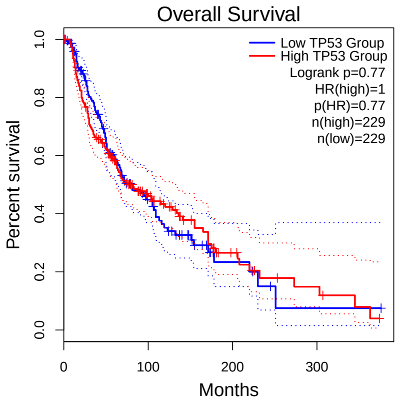

(b) Overall Survival of SKCM

Figure S13: Overall Survival

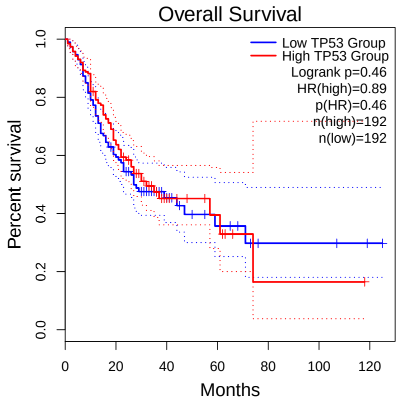

(a) Overall Survival of STAD

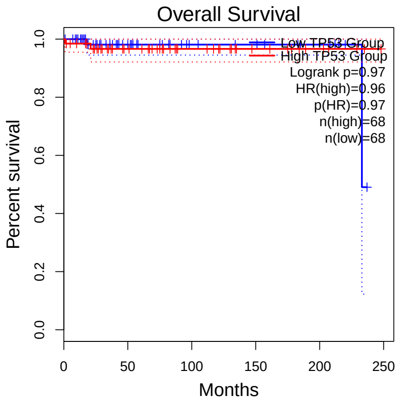

(b) Overall Survival of TGCT

Figure S14: Overall Survival

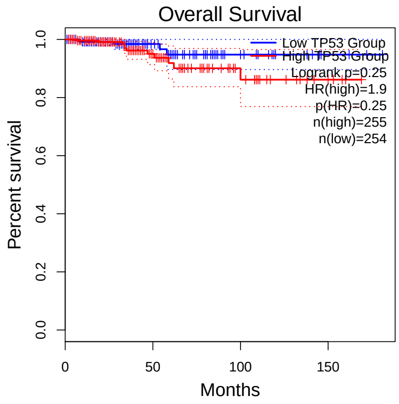

(a) Overall Survival of THCA

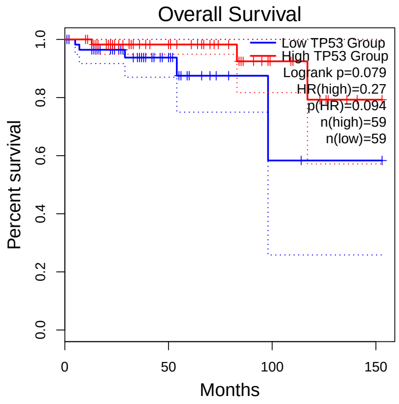

(b) Overall Survival of THYM

Figure S15: Overall Survival

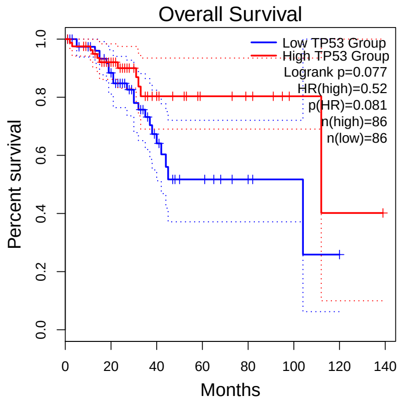

(a) Overall Survival of UCEC

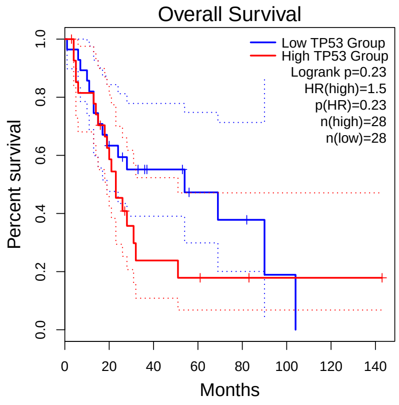

(b) Overall Survival of USC

Figure S16: Overall Caption for both Images

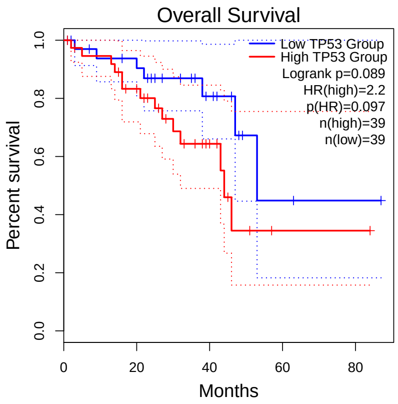

(a) Overall Survival of UVM
