# Supplementary material for: Comprehensive Bioinformatic Investigation of TP53 Dysregulation in Diverse Cancer Landscapes
Source: Genes (Basel). 2024 Apr 30;15(5):577. doi: 10.3390/genes15050577 (PMC11121236; doi:10.3390/genes15050577)
Supplement: Supplementary file 1 [file genes-15-00577-s001.zip › genes-2922551-supplementary file S3-DFS.pdf]

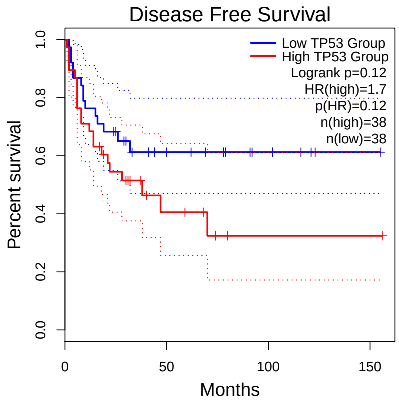

(a) Disease Free Survival (RFS) for ACC

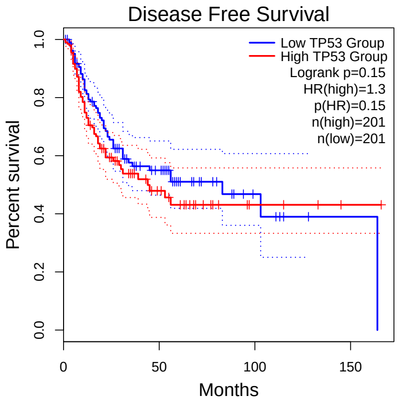

(b) Disease Free Survival (RFS) for BLCA

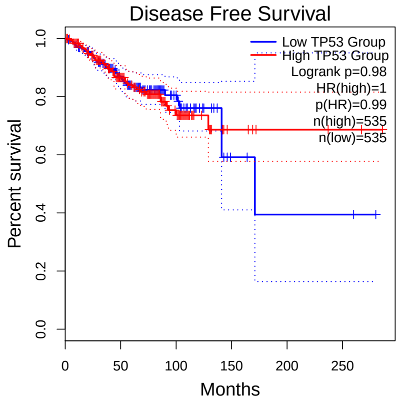

(a) Disease Free Survival (RFS) for BRCA

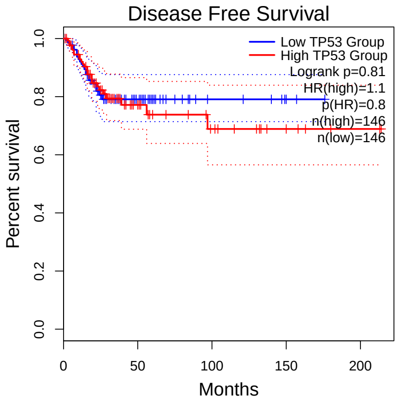

(b) Disease Free Survival (RFS) for CESC

V

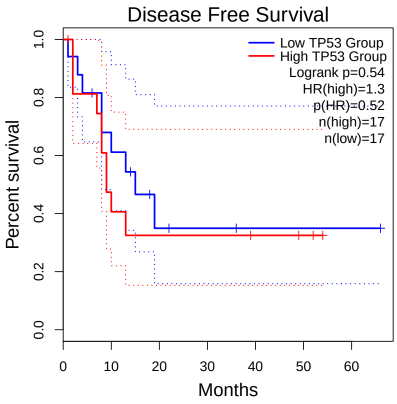

(a) Disease Free Survival (RFS) for CHOL

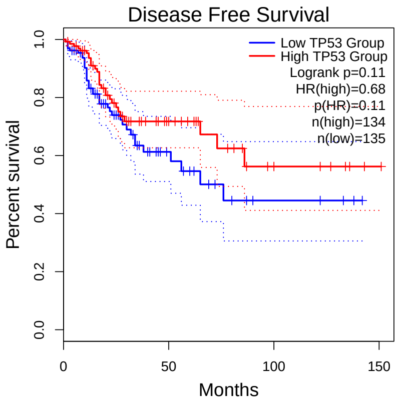

(b) Disease Free Survival (RFS) for COAD

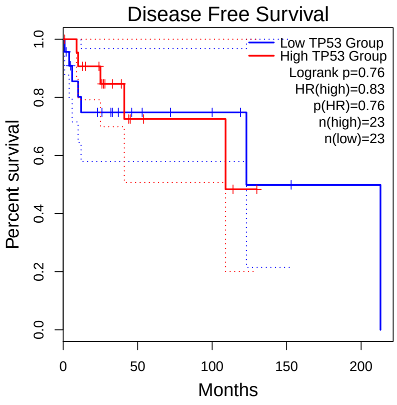

(a) Disease Free Survival (RFS) for DLBC

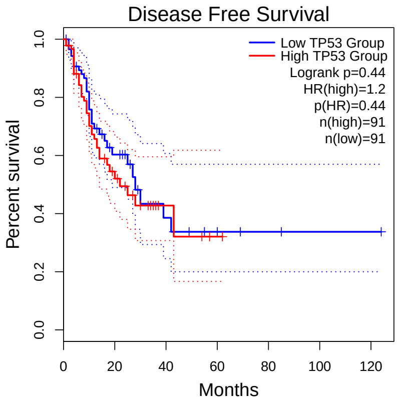

(b) Disease Free Survival (RFS) for ESCA

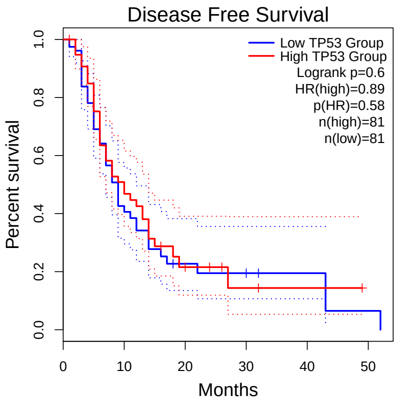

(a) Disease Free Survival (RFS) for GBM

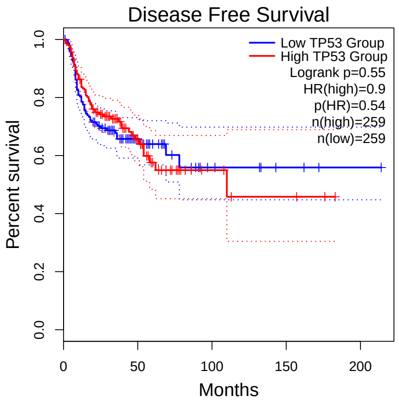

(b) Disease Free Survival (RFS) for HNSC

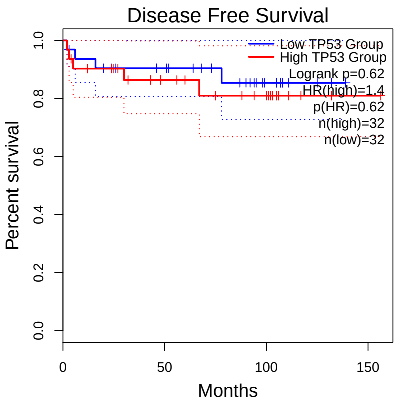

(a) Disease Free Survival (RFS) for KICH

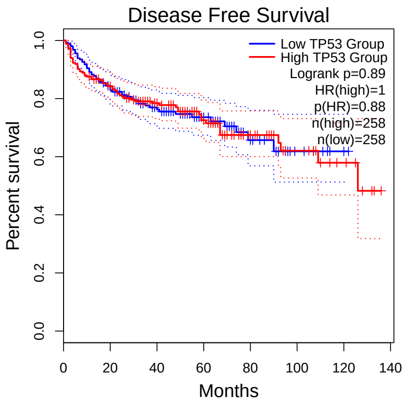

(b) KIRC

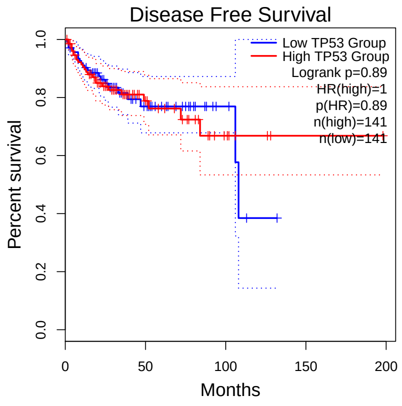

(a) Disease Free Survival (RFS) for KIRP

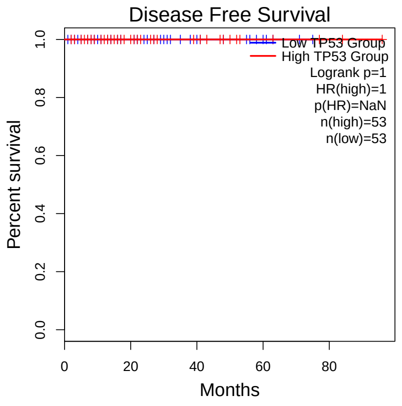

(b) Disease Free Survival (RFS) for LAML

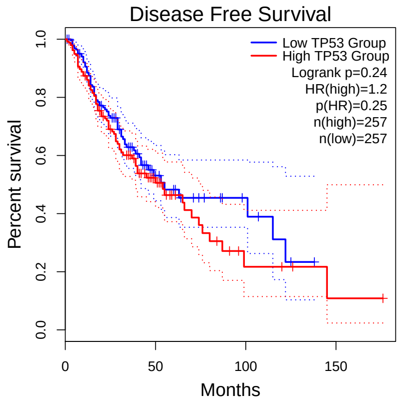

(a) Disease Free Survival (RFS) for LGG

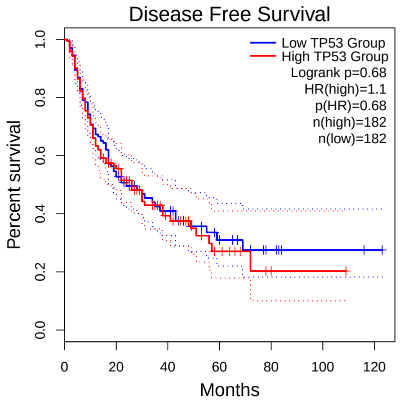

(b) LIHC

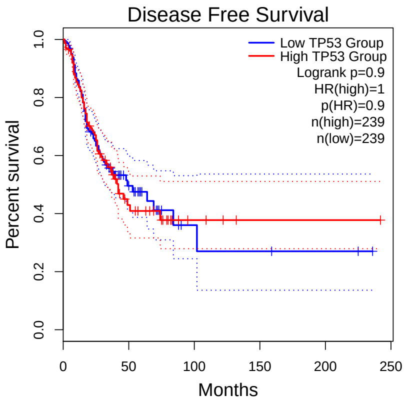

(a) Disease Free Survival (RFS) for LUAD

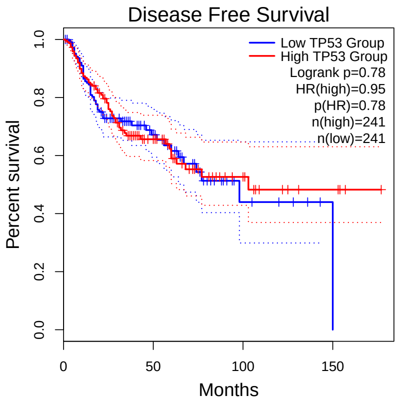

(b) Disease Free Survival (RFS) for LUSC

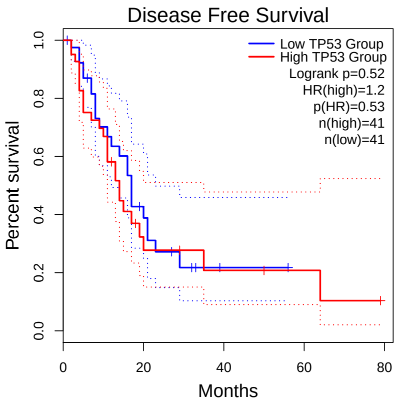

(a) Disease Free Survival (RFS) for MESO

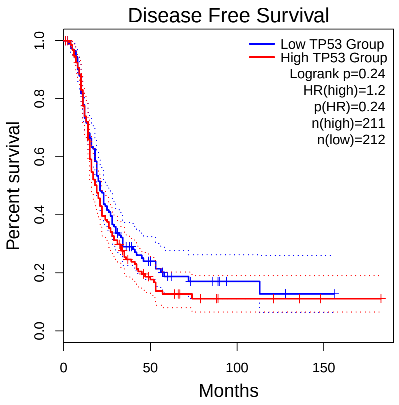

(b) Disease Free Survival (RFS) for OV

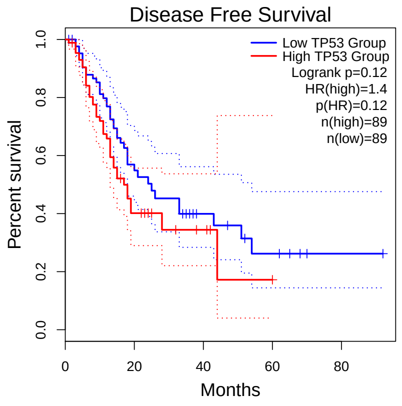

(a) Disease Free Survival (RFS) for PAAD

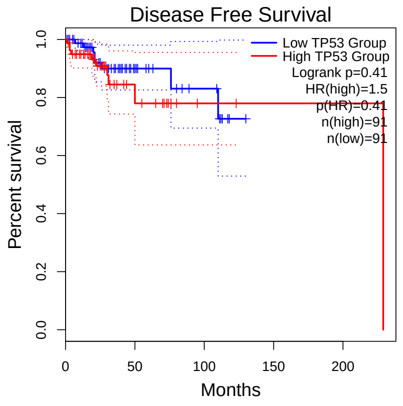

(b) Disease Free Survival (RFS) for PCPG

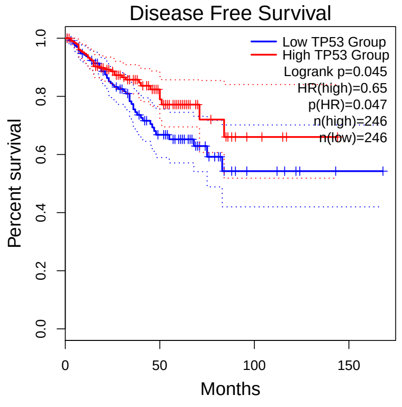

(a) Disease Free Survival (RFS) for PRAD

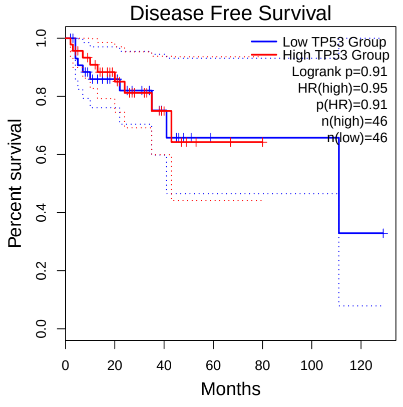

(b) Disease Free Survival (RFS) for READ

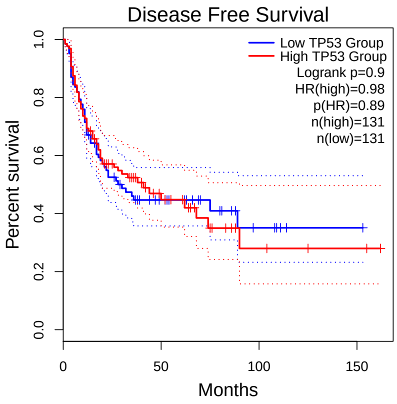

(a) Disease Free Survival (RFS) for SARC

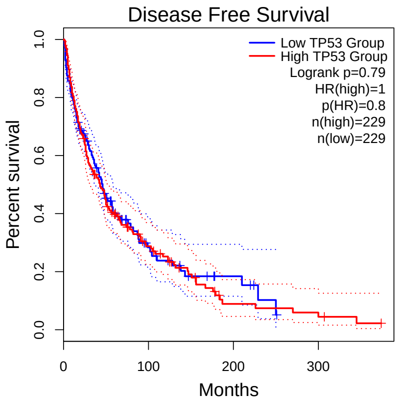

(b) Disease Free Survival (RFS) for SKCM

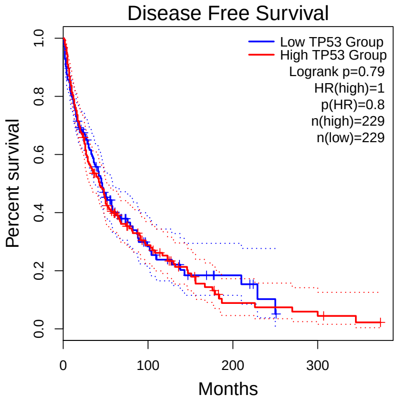

(a) Disease Free Survival (RFS) for STAD

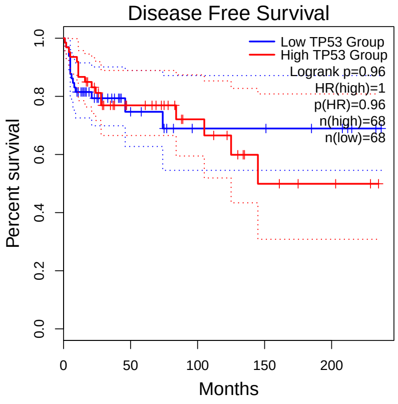

(b) Disease Free Survival (RFS) for TGCT

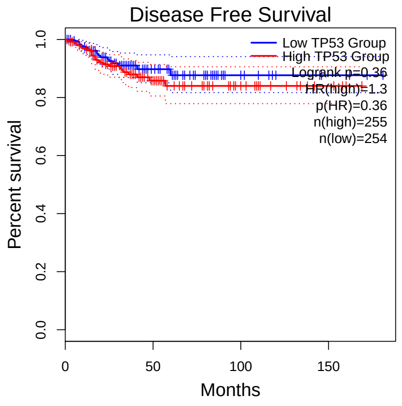

(a) Disease Free Survival (RFS) for THCA

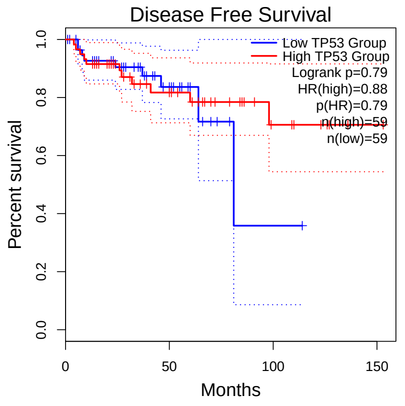

(b) Disease Free Survival (RFS) for THYM

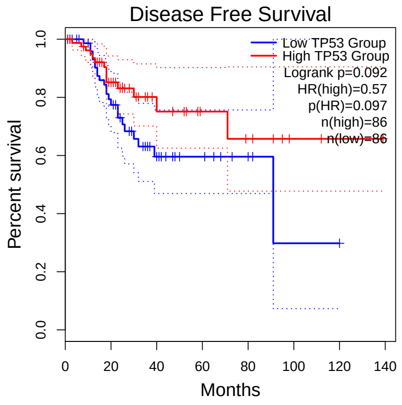

(a) Disease Free Survival (RFS) for UCEC

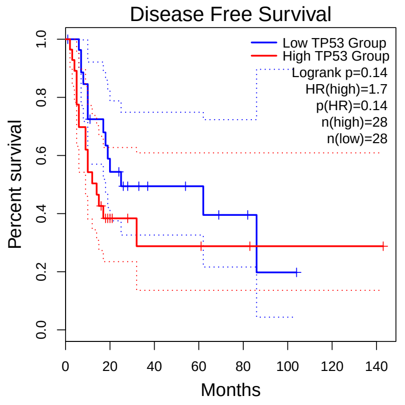

(b) Disease Free Survival (RFS) for UCS

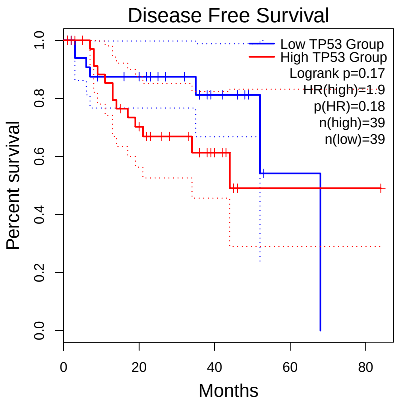

(a) Disease Free Survival (RFS) for UVM
